# Experiences of adolescents with type 1 diabetes, their parents, and health care providers during the transition to adult health care settings: a qualitative systematic review

**DOI:** 10.11124/JBIES-24-00507

**Published:** 2025-12-23

**Authors:** Mariam G. Ahmed, Emma Wilson, Heather Buchanan, Fatin Pakururazi, James Law, Jo Leonardi-Bee

**Affiliations:** 1 Nottingham Centre for Public Health and Epidemiology, School of Medicine, University of Nottingham, Nottingham, UK; 2 Public Health and Community Medicine Department, Faculty of Medicine, Assiut University, Assiut, Egypt; 3 Nottingham JBI Centre for Evidence-Based Healthcare, Nottingham, UK; 4 Department of Pharmacy Practice and Policy, School of Pharmacy, University of Nottingham, Nottingham, UK; 5 Department of Clinical Pharmacy and Pharmacy Practice, Faculty of Pharmacy, University of Sultan Zainal Abidin, Terengganu, Malaysia; 6 Nottingham University Hospitals NHS Trust, Nottingham, UK

**Keywords:** health care transition, adolescent health, type 1 diabetes, qualitative research

## Abstract

**Objective:**

The objective of this qualitative review was to identify and synthesize the evidence related to the experiences and perspectives of adolescents with type 1 diabetes (T1D), their parents, and health care providers (HCPs) on the transition from pediatric to adult health care settings.

**Introduction:**

Transition to adult health care is one of the challenges facing adolescents with T1D, and unsuccessful transitions can lead to negative health outcomes. Understanding the perspectives of adolescents and young adults, their parents, and health care professionals on the transition is essential to develop effective transition interventions.

**Eligibility criteria:**

Studies were considered for inclusion if they explored the experiences and perspectives of adolescents with T1D, their parents, and HCPs on the transition from pediatric to adult health care settings. This review included studies that focused on qualitative data, including designs such as phenomenology, grounded theory, ethnography, and qualitative descriptive.

**Methods:**

MEDLINE (Ovid), Embase (Ovid), PsycINFO (Ovid), CINAHL (EBSCOhost), Cochrane Library, and PubMed were searched to identify published studies from inception to May 2024. There was no restriction on the date or language of the publication. The conduct of the review adhered to the JBI methodology for qualitative systematic reviews. The JBI process of meta-aggregation was used to identify categories and synthesize findings.

**Results:**

Of 6303 records identified, 25 studies met the eligibility criteria and were included in the review. The included studies represented the perspectives of 486 adolescents and young adults receiving care in either pediatric or adult health care settings, 86 parents, and 81 HCPs working in either pediatric or adult health care settings. In most studies, interviews were used as the method for data collection. A total of 159 findings were extracted and aggregated into 12 categories, based on the similarity of meaning. Five synthesized findings were generated, all with moderate confidence: i) Adolescents and young adults with T1D may be anxious and feel unprepared due to being unaware of the process and a lack of preparation; however, others may be confident and ready for their transition to adult services; ii) Adolescents with T1D face many barriers as they transition from pediatric to adult care, mainly due to challenges of navigating changes in health systems or the burden of living with T1D; iii) A successful transition to adult care for adolescents and young adults with T1D depends on activities to support feeling prepared for the transition, having support networks with a stronger emphasis on peer support, and a structured and coordinated transition between pediatric and adult health care; iv) Parents can struggle with their child’s transition where the parents’ roles and responsibilities are refined by their children as they approach adulthood, and parents may need support to address their new role during transition; and v) HCPs can be uncertain of the best transition strategy and tend to suggest many activities to improve transition, mainly focusing on coordination between pediatric and adult health care.

**Conclusion:**

The findings of this qualitative review offer insight into the perspectives of adolescents with T1D, parents, and HCPs on the transition from pediatric to adult health care settings. The findings highlighted the need for more structured transition processes that empower autonomy and self-management skills of young adults, along with continuous support from parents and HCPs.

**Review registration:**

PROSPERO CRD42024557117

**Table:** ConQual Summary of Findings

Experiences of adolescents with type 1 diabetes, their parents, and health care providers during the transition to adult health care settings					
Bibliography: Ahmed MG, Wilson E, Buchanan H, Pakururazi F, Law J, Leonardi-Bee J. Experiences of adolescents with type 1 diabetes, their parents, and health care providers during the transition to adult health care settings: a qualitative systematic review. JBI Evid Synth. 2026;24(1):77-141.					
Synthesized finding	Type of research	Dependability	Credibility	ConQual score	Comment
Adolescents and young adults with T1D may be anxious and feel unprepared due to being unaware of the process and a lack of preparation; however, others may be confident and ready for their transition to adult services.	Qualitative	Moderate (downgraded 1 level)	High (no change)	Moderate	Dependability: Moderate due to a mix of studies that scored 4–5 or 2–3 for the questions relating to appropriateness of the conduct of the research (8 moderate and 3 high)
					Credibility: High, as most findings are unequivocal (U=25, C=1)
Adolescents with T1D face many barriers as they transition from pediatric to adult care, mainly due to challenges of navigating changes in health systems or the burden of living with T1D.	Qualitative	Moderate (downgraded 1 level)	High (no change)	Moderate	Dependability: Moderate due to a mix of studies that scored 4–5 or 2–3 for the questions relating to appropriateness of the conduct of the research (10 moderate and 4 high)
					Credibility: High, as most findings are unequivocal (U=48, C=2)
A successful transition to adult care for adolescents and young adults with T1D depends on activities to support feeling prepared for the transition, having support networks with a stronger emphasis on peer support, and a structured and coordinated transition between pediatric and adult health care.	Qualitative	Moderate (downgraded 1 level)	High (no change)	Moderate	Dependability: Moderate due to a mix of studies that scored 4–5 or 2–3 for the questions relating to appropriateness of the conduct of the research (10 moderate and 5 high)
					Credibility: High, as most findings are unequivocal (U=47, C=1)
Parents can struggle with their child’s transition where the parents’ roles and responsibilities are refined by their children as they approach adulthood, and parents may need support to address their new role during transition.	Qualitative	Moderate (downgraded 1 level)	High (no change)	Moderate	Dependability: Moderate due to a mix of studies that scored 4–5 or 2–3 for the questions relating to the appropriateness of the conduct of the research (3 moderate and 1 high)
					Credibility: High, as most findings are unequivocal (U=16, C=1)
Health care providers can be uncertain of the best transition strategy and tend to suggest many activities to improve transition, mainly focusing on coordination between pediatric and adult health care.	Qualitative	Moderate (downgraded 1 level)	High (no change)	Moderate	Dependability: Moderate due to mix of studies scored 4-5 or 2-3 for the questions relating to appropriateness of the conduct of the research (4 moderate and 1 high)
					Credibility: High, as most findings are unequivocal (U=23, C=1)

Note: The number of findings in the ConQual summary is higher than in the abstract and main text because findings with multiple illustrations across different categories were counted only once in the the abstract and text, but expanded in the ConQual summary

U, unequivocal; C, credible; T1D, type 1 diabetes

## Introduction

Type 1 diabetes (T1D) in children, adolescents, and young adults is a chronic autoimmune disease that requires daily care to prevent both acute and chronic complications. T1D can develop at any age; however, the peak incidence occurs during puberty in children 10 to 14 years old.^1^-^4^ The incidence of T1D among young children has continued to increase worldwide over the past few decades,^2^,^5^ with approximately 108,300 children under 15 years of age estimated to be diagnosed with T1D every year globally.^6^ The growing incidence represents a challenge to health care systems in managing the transition of these adolescents with diabetes from pediatric to adult diabetes units.^7^

Many health care systems transfer pediatric patients to adult care at 18 years of age, which can also be a time of major change in school, work status, and residence of the individual.^8^ Additionally, the young adult is leaving what has often been a long-term, trusting relationship with a health care provider (HCP), and this departure can sometimes occur without the necessary preparation or access to a subsequent provider.^9^ Young adults who are abruptly transferred from pediatric to adult care are more likely to have poor glycemic control than those who stayed with their pediatric provider.^10^-^12^ In the adult care setting, a young adult with T1D who is newly transferred is often expected to be wholly responsible for their treatment regimen, make autonomous health care decisions, and interact with their HCPs independently^13^; therefore, the young adult often needs to establish and maintain daily self-care routines that include administering treatments, testing blood glucose levels, and treating occurrences of hypoglycemia (low blood sugar).^14^

It is estimated that 30% of young adults with T1D disengage from care during the transition period, with approximately half reporting difficulties with the transition process as the reason for disengagement.^15^-^17^ Disengagement can result in a decline in clinic attendance,^18^,^19^ with serial non-attendees having poorer glycemic control than those who remain in medical care.^20^ The consequences of suboptimal glycemic control in young adults with T1D are increased risks of hospitalization and diabetes-related complications, resulting not only in an increased burden on health care systems but also in significant impacts on daily activities, work, relationships, and overall quality of life.^16^,^21^,^22^

All these challenges and difficulties demonstrate the critical need for effective programs to support the transition of care from pediatric to adult health settings. Several professional societies have proposed that purposeful planning for the health care transition can prevent discontinuities in specialized care, develop independent self-management and self-advocacy, and reduce adverse health outcomes in this population.^9^,^23^,^24^ Many transition care strategies have been described in the literature, and generally, 3 broad categories of interventions have been evaluated: i) patient-focused (educational programs, skills training); ii) staff-focused (having named transition coordinators, joint clinics run by pediatric and adult physicians); and iii) service delivery–focused (having separate clinics, out-of-hours phone support, or enhanced follow-up for young adults).^20^,^25^-^30^

Understanding the perspectives of young adults, their parents, and HCPs on the transition from pediatric to adult care for adolescents with T1D is essential to aid the development of effective transition interventions. A preliminary search was conducted in MEDLINE, Embase, Cochrane Library, and PROSPERO, and no other current or in-progress systematic reviews on the topic were found. The aim of this qualitative systematic review was to identify and synthesize findings on how adolescents and young adults with T1D, their parents, and HCPs experience the adolescent’s or young adult’s transition from pediatric to adult health care settings.

## Review questions

- What are the experiences and perspectives of adolescents with T1D, their parents, and HCPs regarding the transition from pediatric to adult health care settings?
- What are the identified barriers and facilitators of the transition from pediatric to adult health care settings?
- What are the expectations and recommendations to improve the transition?

## Eligibility criteria

The eligibility criteria were developed using the PICo (participants, pheomena of interest, context) framework.

### Participants

This review considered studies including adolescents and young adults with T1D, defined as those aged 15 years to their late 20s (young adults were included to capture retrospective experiences of the transition to adult care); their parents; or HCPs working in diabetic health care.

### Phenomena of interest

Studies were considered for inclusion if they explored the experiences and perspectives of adolescents and young adults with T1D, their parents, and/or HCPs regarding the young person’s transition to adult health care settings. While both experiences and perspectives are interlinked, we define *experiences* as a result of directly encountering and engaging in a phenomenon of interest; in contrast, we define *perspectives* as being shaped by past experiences, beliefs, and values, and how we interpret experiences.

Studies were excluded if their focus was type 2 diabetes, different chronic diseases, or the general transition to adulthood; if T1D was not specified in the results; or if the focus was not specific to the transition to adult health care.

### Context

Diabetes clinics in any health care setting globally were considered eligible for inclusion.

### Types of studies

This review included studies that focused on qualitative data, including designs such as phenomenology, grounded theory, ethnography, and qualitative descriptive. Mixed methods studies were also included where data could be extracted from the qualitative component of the study. Studies were excluded if they were abstracts or conference proceedings, as they lack depth and do not provide sufficient detail to capture all relevant themes and illustrations needed for a comprehensive analysis. Book chapters, dissertations, and other non-peer-reviewed sources were also excluded.

## Methods

The proposed systematic review was conducted in accordance with the JBI methodology for systematic reviews of qualitative evidence^31^ and reported according to the Preferred Reporting Items for Systematic Reviews and Meta-Analysis (PRISMA)^32^ and the Enhancing Transparency in Reporting the Synthesis of Qualitative Research (ENTREQ)^33^ guidelines. The protocol for the systematic review was registered with PROSPERO (CRD42024557117). There were no deviations from the methods outlined in the a priori protocol.

### Search strategy

An initial limited search of MEDLINE (Ovid) and Embase (Ovid) was undertaken to identify articles on the topic. The text words contained in the titles and abstracts of relevant articles, and the index terms used to describe the articles were used to develop a full search strategy for MEDLINE. The search strategy, including all identified keywords and index terms, was adapted for each included information source. The search strategy included terms for T1D and transition to adult health care services (Appendix I). The reference lists of all included articles and relevant systematic reviews were manually screened for additional studies. Titles and abstracts of studies not available in English were first translated using Google Translate to determine their eligibility. For relevant non-English studies, full-text translations were sought using DeepL (DeepL, Cologne, Germany).

The following 6 databases were searched for published studies from their inception to March 2023, and updated in May 2024: MEDLINE (Ovid), Embase (Ovid), PsycINFO (Ovid), CINAHL (EBSCOhost), Cochrane Library (Cochrane Database of Systematic Reviews and CENTRAL), and PubMed.

There were no restrictions on the date or language of the publication.

### Study selection

Following the searches, all identified citations were collated and uploaded into Mendeley 1.19.5 (Mendeley Ltd., Elsevier, Netherlands), and deduplicated using Mendeley’s built-in tool. Rayyan.ai (Qatar Computing Research Institute, Doha, Qatar) was used for rechecking the duplication. Two reviewers (MA, EW) conducted a pilot test of 10% of the eligible titles and abstracts (agreement=95%). The remaining titles and abstracts and full texts were screened independently by the same 2 reviewers in Rayyan.ai.

Any disagreements were resolved through discussion or with a third reviewer (HB). All decisions were agreed upon following discussion.

### Assessment of methodological quality

Eligible studies were critically appraised by 2 reviewers (MA, JLB) independently for methodological quality using the JBI checklist for qualitative research.^34^ Each paper was assigned a percentage score out of a total score of 10 based on whether each domain was met by assigning a score to each question, where 1 point was given for “yes,” 0.5 for “unclear,” and 0 for “no.” Any disagreements between the reviewers were resolved through discussion. All studies underwent data extraction and synthesis regardless of their quality.

### Data extraction

Data were extracted from studies by 2 reviewers (MA, FP) using the standardized JBI data extraction tool for qualitative reviews.^34^ Authors of the primary studies were contacted for clarification or to obtain missing information. The data extracted included the aim of study; data collection process; method of analysis; sample size; context (country in which the study was conducted); process of recruitment; and the participants’ gender, age range, age of transition to adult health care setting, and duration of diabetes. Additionally, the findings of the study were extracted using themes or subthemes together with an illustrative quote to represent that finding. Two reviewers (MA, FP) independently rated each extracted finding and assigned a level of credibility: unequivocal (findings with illustrations beyond reasonable doubt and, thus, not open to challenge); credible (findings with illustrations that are plausible and inferred from the data, but open to challenge); or not supported (findings not supported by the data. Consensus was reached through discussion).

### Data synthesis

Qualitative data were, where possible, pooled using the JBI qualitative evidence synthesis approach^31^ and presented in tabular format. The process involved the aggregation or synthesis of findings to generate a set of statements representing that aggregation, through assembling the findings and categorizing these findings based on similarity in meaning/wording.^31^ These categories were then subjected to synthesis to produce a single comprehensive set of synthesized findings that could be used as a basis for evidence-based practice. Only unequivocal and credible findings were included in the synthesis.

It happened in some studies that certain findings were reported by more than one group; for example, by both adolescents and parents, or by adolescents and HCPs. These findings with multiple illustrations ended up appearing across different categories.

### Assessing confidence in the findings

The final synthesized findings were graded according to the ConQual approach for establishing confidence in the output of qualitative research synthesis and presented in a Summary of Findings.^35^ The Summary of Findings includes the major elements of the review and details how the ConQual score was developed. Each synthesized finding from the review is presented, along with the type of research informing it, scores for dependability and credibility, and the overall ConQual score.

## Results

### Study inclusion

**Figure 1:**
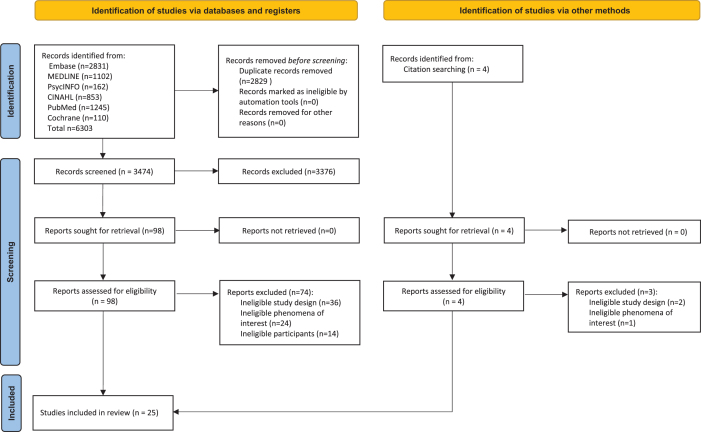
Search strategy and study selection and inclusion process^32^

Out of 3474 deduplicated hits from the search, 98 studies were deemed potentially eligible based on title and abstract screening. Of these, 25 studies were included in the review, including 1 study identified from scanning the reference lists. The remaining 74 studies were excluded at the full-text stage due to ineligible study design (36 studies), ineligible phenomena of interest (24 studies), or ineligible participants (14 studies). Figure 1 shows the search results, and study selection and inclusion process. The complete list of studies excluded at full-text screening is provided in Appendix II.

### Methodological quality

Overall, the methodological quality of the 25 included studies was high (Table 1), with 1 paper having an overall score of 100%,^36^ 2 papers with an overall score of 95%,^37^,^38^ 4 papers with an overall score of 90%,^13^,^39^-^41^ 3 papers with overall score of 85%,^42^-^44^ 4 papers with overall score of 80%,^45^-^48^ 7 papers with an overall score 75%,^49^-^55^ 3 papers with an overall score 70%,^56^-^58^ and 1 paper with an overall score of 65%.^59^ Nearly all studies demonstrated congruities between the research methodology and research objectives, data collection methods, data representation and analysis, and interpretation of results. There were concerns in some studies regarding stating the researcher’s philosophical perspective, locating the researcher culturally or theoretically, and addressing the researcher’s influence on the research. Most studies showed that the research was ethical, and all studies had conclusions that flowed from the analysis or interpretation of the data. All studies but one had sufficient representation of participants’ voices; this study was not included in meta-aggregation, as its findings were not supported.^59^

**Table 1:** Critical appraisal of eligible qualitative studies

Author, year	Q1	Q2	Q3	Q4	Q5	Q6	Q7	Q8	Q9	Q10	%
Allen *et al.*,^52^ 2011	U	Y	Y	Y	Y	N	N	Y	Y	Y	75
Butalia *et al.*,^47^ 2020	U	Y	Y	Y	Y	N	U	Y	Y	Y	80
Garvey *et al.*,^42^ 2014	U	Y	Y	Y	Y	N	Y	Y	Y	Y	85
Guitard-Munnich and Houdan,^57^ 2019	U	Y	Y	Y	Y	N	N	Y	U	Y	70
Hansen and Jensen,^58^ 2017	U	Y	Y	Y	Y	N	N	Y	U	Y	70
Harris *et al.*,^39^ 2020	U	Y	Y	Y	Y	U	Y	Y	Y	Y	90
Hilliard *et al.*,^44^ 2014	U	Y	Y	Y	Y	U	U	Y	Y	Y	85
Iversen *et al.*,^36^ 2019	Y	Y	Y	Y	Y	Y	Y	Y	Y	Y	100
Ladd *et al.*,^43^ 2022	U	Y	Y	Y	Y	U	U	Y	Y	Y	85
Laursen *et al.*,^38^ 2024	Y	Y	Y	Y	Y	Y	U	Y	Y	Y	95
Leung *et al.*,^50^ 2021	U	Y	Y	Y	Y	N	N	Y	Y	Y	75
Lundin *et al.*,^40^ 2007	Y	Y	Y	Y	Y	U	U	Y	Y	Y	90
McDowell et al.,^46^ 2020	U	Y	Y	Y	Y	N	U	Y	Y	Y	80
Nakhla *et al.*,^59^ 2017	U	Y	Y	Y	Y	N	N	N	Y	Y	65
Olsson *et al.*,^37^ 2023	U	Y	Y	Y	Y	Y	Y	Y	Y	Y	95
Perry *et al.*,^13^ 2012	U	Y	Y	Y	Y	U	Y	Y	Y	Y	90
Pritlove *et al.*,^41^ 2020	U	U	Y	Y	Y	Y	Y	Y	Y	Y	90
Pyatak *et al.*,^54^ 2014	U	Y	Y	Y	Y	N	N	Y	Y	Y	75
Ritholz *et al.*,^45^ 2014	U	Y	Y	Y	Y	N	U	Y	Y	Y	80
Simms *et al.*,^56^ 2017	U	Y	Y	Y	Y	N	N	Y	U	Y	70
Tremblay *et al.*,^49^ 2020	U	Y	Y	Y	Y	N	U	Y	U	Y	75
Vaillancourt *et al.*,^48^ 2023	U	Y	Y	Y	Y	N	U	Y	Y	Y	80
Visentin *et al.*,^51^ 2006	U	Y	Y	Y	Y	N	N	Y	Y	Y	75
Walsh *et al.*,^55^ 2018	U	Y	Y	Y	Y	N	N	Y	Y	Y	75
Williams *et al.*,^53^ 2021	U	Y	Y	Y	Y	N	N	Y	Y	Y	75

Y, yes; N, no; U, unclear.

JBI Critical Appraisal Checklist for Qualitative Research^34^

Q1: Is there congruity between the stated philosophical perspective and the research methodology?

Q2: Is there congruity between the research methodology and the research question or objectives?

Q3: Is there congruity between the research methodology and the methods used to collect data?

Q4: Is there congruity between the research methodology and the representation and analysis of data?

Q5: Is there congruity between the research methodology and interpretation of results?

Q6: Is there a statement locating the researcher culturally or theoretically?

Q7: Is the researcher’s influence on the research addressed?

Q8: Are participants, and their voices, adequately represented?

Q9: Is the research ethical according to current criteria, or for recent studies, is there evidence of ethical approval by an appropriate body?

Q10: Do the conclusions drawn from the research report flow from the analysis or interpretation of the data?

### Characteristics of the included studies

The 25 studies represented the perspectives of 486 adolescents and young adults receiving care in either pediatric or adult health care settings, 86 parents, and 81 HCPs working in either pediatric or adult health care settings (Appendix III). Ten studies explored expectations/experiences/concerns of adolescents before the transition to adult health care setting,^43^,^44^,^47^,^49^-^52^,^55^-^57^ 13 studies investigated experiences/perceived facilitators and barriers of young adults after the transition to adult health care settings,^13^,^36^-^38^,^42^,^44^-^48^,^54^,^57^,^58^ 5 studies investigated parents’ perspectives regarding the transition,^41^,^44^,^47^,^52^,^55^ and 6 studies explored HCPs’ perspectives regarding transition.^39^,^40^,^51^,^53^,^55^,^59^

Seven studies were conducted in the USA,^42^,^44^-^46^,^49^,^54^,^56^ 7 studies were conducted in Canada,^41^,^43^,^47^,^48^,^50^,^53^,^59^ 3 studies in Australia,^13^,^39^,^51^ 2 studies in Sweden,^37^,^40^ 2 studies in Denmark,^38^,^58^ and single studies were conducted in Norway,^36^ the UK,^52^ Ireland,^55^ and France.^57^ Only 1 study was published in French,^57^ and the full text was translated to English using the online translator DeepL. Data were collected by semi-structured interviews in most of the studies,^36^,^38^-^41^,^44^,^48^,^49^,^53^-^58^ with a further 4 studies using focus group discussions,^42^,^45^,^47^,^50^ 1 study using online surveys,^46^ and 1 study using both participant observation and interviews.^40^ Although transition practices were described in some studies, the majority did not report having an established structured transition care program.

### Review findings

A total of 159 findings were extracted from the 25 included studies, with 153 rated as unequivocal and 6 rated as credible (Appendix IV). A further 16 were not supported and not included in the meta-synthesis. The 159 findings assigned as credible or unequivocal were aggregated into 12 categories and resulted in the following 5 synthesized findings (Appendix V) and assigned a ranking for the confidence in the findings (see the Summary of Findings).

#### Synthesized finding 1: Adolescents and young adults with T1D may be anxious and feel unprepared due to being unaware of the process and a lack of preparation; however, others may be confident and ready for their transition to adult services

This synthesized finding was assigned moderate confidence according to ConQual and relates to the perceptions of adolescents and young adults with T1D regarding their transition to adult health care. It includes 3 categories derived from 26 findings identified in 11 studies.^36^,^37^,^42^-^44^,^48^,^49^,^55^-^58^

##### Category 1.1: Transition preparedness and associated anxiety

This category was derived from 10 findings identified in 6 studies.^36^,^37^,^42^,^43^,^48^,^49^ Most adolescents in these studies reported receiving minimal information regarding their transition, and they consistently felt they needed more guidance and support from their HCPs during and after transition.

*I’d say that I definitely didn’t understand all of my options and um I feel like it’s made it difficult since leaving, and also like, if I wanted to change my option now from being at a private clinic to going to a hospital, I wouldn’t even know how to go about that. I don’t want to that, I’m just saying, but if I did want to do that, um I don’t know, I just feel like it was the last appointment that we talked about it and by then it’s too late cause I’m not seeing my doctor again. If I was to change doctors I feel like it should be done almost a year in advance. That would, for sure make the process much easier. (Male, 19 years)*^48^^(Suppl 2)^

Some adolescents expressed anxiety and avoidance of the topic of transition.

*I don’t want to talk about it…. I just turned 18, so I don’t want to leave yet. (Female, 18 years)*^49^^(p.334)^

##### Category 1.2: Unawareness of the transition process

This category was derived from 5 findings identified in 3 studies.^55^,^57^,^58^ Adolescents were generally unaware of what the process of transition entails. Indeed, they did not even realize they were part of the process of transition, and that they could influence this process. Rather, they considered it as a physical move or an inevitable step in their health care, concurrent with their age and developmental stage.

*I think it was just that, you get old enough, go to the adult one…simple as that. (A young adult)*^55^^(p.787)^

It’s inevitable. (A young adult)^57^^(p.414)^

##### Category 1.3: Confidence to transition

This category was derived from 11 findings identified in 6 studies.^37^,^43^,^44^,^48^,^55^,^56^ Some participants believed that the age of diagnosis had an impact on their ability to manage diabetes, and a longer diabetes duration made them more comfortable with self-management and ready for transition to adult care.

*I felt like I really just, like, as a person matured more, and like, taking care of myself, it like, really like, coincided with me becoming an adult, especially in my diabetes development, I became more mature, knowing how to handle myself for my own good and just taking more initiatives, so I’ve definitely, ya, I think it’s worked out fine. (Participant 2, male, age=18 years)*^48^^(Suppl 2)^

Others, interestingly, described the positive aspects of leaving pediatric care, such as being more accountable or involved, taking on greater responsibility and control over diabetes management and not feeling embarrassed to ask about smoking and drugs without fear of being judged.

*I feel much more comfortable talking to her about pretty much anything; like ‘hey, I’m having a problem with this, and it’s kind of personal and embarrassing, but it’s an issue’. (Young adult)*^56^^(p.420)^

#### Synthesized finding 2: Adolescents with T1D face many barriers as they transition from pediatric to adult care, mainly due to challenges of navigating changes in health systems or the burden of living with T1D

This synthesized finding was assigned moderate confidence according to ConQual and relates to barriers of transition to adult health care. This synthesized finding was derived from 50 findings from 2 categories in 14 studies.^13^,^36^,^37^,^42^,^43^,^45^,^46^,^48^-^50^,^54^,^55^,^57^,^58^

##### Category 2.1: Health system challenges

This category was derived from 25 findings identified in 13 studies.^13^,^36^,^37^,^42^,^43^,^45^,^46^,^48^-^50^,^54^,^57^,^58^ Many adolescents were sad to leave the pediatric clinic and end the long-term relationship with their pediatrician.

*I’m actually kind of sad that I’m leaving the team, they were so great. (Participant 12, an adolescent)*^43^^(p.6)^

Many post-transition adolescents described differences between pediatric and adult health care settings. They described the pediatric care as a safe, comfortable environment with more frequent follow-up and better multidisciplinary care, while the adult care as less organized with less personal support, less frequent follow-ups, separation of services, and shorter consultations that could not address all their needs.

*The most shocking thing was coming in the first time, and it wasn’t the standard procedure I was used to. Yeah, it’s just something you have to kind of pick up […]. It was just very different. (female, age 25)*^42^^(p.194)^

Many young adults encountered challenges in the public health system after being discharged from pediatric care, including long waiting times, misinformation, difficulty navigating the ins and outs of insurance, and the bureaucracy of the public health system.

*I called for an appointment to see a doctor…They said that they charged every time you go to an appointment… Eventually, we had to go to the emergency room to get insulin. And they were like, “So why didn’t you go to a doctor [at County]?” And I told them that they wanted to charge me. And they said, no, that is not true, that it is free. (Male, a young adult)*^54^^(p 1618)^

##### Category 2.2: Burden of living with T1D

This category was derived from 25 findings identified in 8 studies.^37^,^42^,^43^,^46^,^50^,^54^,^55^,^57^ Many participants reported difficulty in adapting to new responsibilities to manage diabetes, especially in light of other competing priorities such as attending college, work caregiving, and family needs.

*When you’re in college, you don’t want to take a day out to travel to Galway to go to a clinic. (a young adult)*^55^^(p.787)^

Some participants expressed a negative attitude toward their disease, feeling stigma and discrimination that interfered with self-care activities (taking medications or checking blood sugar in front of their peers) and made them hesitant to disclose their diagnosis to their friends.

*I perceive myself as more ill when I’m supposed to pick up a cannula, adjust the pen, and do injections in my stomach. I experience myself as sicker. I also believe it to be harder to do this among others, somehow. (Participant 7, a young adult)*^37^^(p.4626)^

Other participants reported struggling with the shifting of responsibility from caregivers to themselves, indicating that some caregivers do not give enough room for autonomy and independence, which are important skills to learn to manage their medical care.

*Not back off cause that’s [kind of] harsh but like a little bit back off … I feel like even if I make mistakes like I’ll learn from them more than if my parents are always [kind of] bothering me about it … [I kind of] need to do that by myself. (Participant 18, an adolescent)*^43^^(p.5)^

#### Synthesized finding 3: A successful transition to adult care for adolescents and young adults with T1D depends on activities to support feeling prepared for the transition; having support networks with a stronger emphasis on peer support; and a structured and coordinated transition between pediatric and adult health care

This synthesized finding was assigned moderate confidence according to ConQual and relates to facilitators of transition to adult health care. This synthesized finding was derived from 48 findings from 3 categories in 15 studies.^13^,^36^-^38^,^42^,^44^-^48^,^50^,^55^-^58^

##### Category 3.1: Preparation of adolescents and young adults for transition to adult health care

This category was derived from 9 findings identified in 7 studies.^13^,^38^,^44^,^46^-^48^,^50^ Many adolescents, in both pre- and post-transition groups emphasized the importance of increased education and early transition preparation to adapt to their upcoming new roles and self-care responsibilities of emerging adulthood.

*…and I know at the Children’s they were like you need to learn to do carb counting but my Mom did everything for me. She still helps me, but I think now, having an educational class to make sure that us as 17-year-olds actually do know how to manage our diabetes on our own.” (group 2, an adolescent)*^47^^(p.4)^

##### Category 3.2: Support of adolescents and young adults for transition to adult health care

This category was derived from 14 findings identified in 10 studies.^13^,^37^,^38^,^44^,^46^-^48^,^50^,^56^,^57^ Many adolescents and parents highlighted the importance of having a social support network with friends who help in accepting the disease and managing it, in addition to interacting with other peers with T1D through social media, diabetes associations, or diabetes camps. They found it very helpful to have someone who could relate to their experience living with diabetes. Some felt that it would be a great source of support to have a peer mentor who had recently gone through transition to adult health care.

*I’d say it might have been helpful to have some sort of I dunno phone number for questions or something or maybe even just I dunno some sort of contact with somebody who has done this before and kind-of knows how it went. (Participant 21, Focus Group 5, an adolescent)*^50^^(p.5)^

Some participants reported they still need to seek support from their parents and HCPs, along with encouraging independence, for a successful transition.

*My parents are just really supportive about that, they want me to get back in control and eating healthy and being portioned. We’re starting carb counting but I’m not 1000% confident with that yet. So I’m just started with portions and doing that. My parents are extremely supportive about that and they’re constantly on my case about it. (Male,19 years)*^48^^(Suppl 2)^

##### Category 3.3: Health care provision improvement

This category was derived from 25 findings identified in 13 studies.^13^,^36^-^38^,^42^,^44^,^45^,^47^,^48^,^50^,^55^,^56^,^58^ Various suggestions for coordination between both pediatric and adult provider teams were voiced by participants, including the presence of a transition coordinator or a common team member, joint clinics, visiting an adult clinic and meeting the adult team prior to transition, specific referral to adult providers and continued role of the pediatrician after transition, and follow-up of participants to ensure success of the transition.

*She [the diabetes nurse] has been with me from the beginning, so she knows that I can manage diabetes… but she also knows that I sometimes fail to manage it. But she knows what can get me on the right track. (Sophie, female, a young adult)*^38^^(p.130)^

*Having appointments where both the pediatric and adult care professionals would be there to meet and discuss my treatment. (21-year-old male)*^44^^(p.351)^

Many participants stressed the importance of having a caring and trusting relationship with their adult HCP, highlighting the desire to be involved in the decision-making processes integral to diabetes care, and considered that an important part of the developmental process of autonomy in diabetes.

*We, who have T1D, come here regularly and the visits are not that personal, so maybe you [i.e., HCPs] could spend a little more time with us. Who we are, what kind of life we are living, and what kind of support we need, and so on. I’d desired that, not simply being a name on a paper.” (Participant 1, a young adult)*^37^^(p.4630)^

Other facilitators mentioned by participants included increased use of communication technology, such as appointment reminders to enhance contact with the diabetes clinic, service flexibility, and provision of specific transition clinics or age-banded clinics.

*I have a lot of flexibility for appointments, they reserve a whole schedule space for students, who of course do not want to miss too much school; I have fixed days but I have a whole schedule of space according to what’s convenient. And When I arrive there, it doesn’t take long; not more than 15 minutes. (Participant 44, female, 19 years)*^48^^(Suppl 2)^

#### Synthesized finding 4: Parents can struggle with their child’s transition where the parents’ roles and responsibilities are refined by their children as they approach adulthood, and parents may need support to address their new role during transition

This synthesized finding was assigned moderate confidence according to ConQual and relates to the parents’ perspective regarding transition of their children with T1D to adult health care. This synthesized finding was derived from 17 findings from 2 categories in 4 studies.^41^,^44^,^52^,^55^

##### Category 4.1: Parental perceived challenges of transition

This category was derived from 10 findings identified in 4 studies.^41^,^44^,^52^,^55^ Parents described significant shifts in their role from the diabetes manager and decision-maker to a more supportive and advisory role. Many emphasized the initial struggle to “let go” despite believing it was the right thing to do.

*I think now the biggest [fear] is that, all through the years you have some sort of control…and now I can’t tell you what his last A1C was. That’s all information that he keeps to himself…That was a big shift, to kind of relinquish control when I had so much control. (P3, a parent)*^41^^(p.4)^

Many parents expressed a feeling of confusion and uncertainty about their role in the new care arrangement. Some felt that they were excluded by HCPs from adult health care settings.

*They kind of don’t encourage us to come. (A parent of a young adult)*^55^^(p.787)^

Other challenges mentioned by parents were lack of structured education, resource limitations, and negative attitude of their children toward their illness.

*He doesn’t give one tuppenny damn about diabetes; he wishes it wasn’t there, he’s kind of angry about it at the moment. (A parent of a young adult)*^55^^(p.787)^

##### Category 4.2: Parental perceived facilitators of transition

This category was derived from 7 findings identified in 3 studies.^44^,^52^,^55^ Many parents encouraged independence and self-management of their children with a level of involvement ensuring that necessary support was in place, whenever needed.

*I’m involved when something is out of the ordinary, if it’s the ordinary she just gets on with it, if she thinks something is a bit different to what she’s expecting then we’ll discuss things with her. (4-M50, a mother)*^52^^(p.997)^

Some parents mentioned some facilitators for transition, such as frequent follow-up, presence of a key person, and having a safety net of social support.

*You always knew you could pick up the phone and ring them… it’s a nice kind of a safety net to have. (A parent of a young adult)*^55^^(p.787)^

#### Synthesized finding 5: Health care providers can be uncertain of the best transition strategy and tend to suggest many activities to improve transition, mainly focusing on coordination between pediatric and adult health care

This synthesized finding was assigned moderate confidence according to ConQual and relates to HCPs’ views regarding transition of children with T1D to adult health care. This synthesized finding was derived from 24 findings from 2 categories in 5 studies.^39^,^40^,^51^,^53^,^55^

##### Category 5.1: HCPs’ perceived challenges of transition

This category was derived from 11 findings identified in 4 studies.^39^,^40^,^51^,^55^ HCPs perceived the current transition process as unstructured and informal.

*At the moment, there’s no follow-up of whether the transition has worked smoothly or not. (A health professional)*^51^^(p.767)^

Some expressed uncertainty about the best transition strategy.

*They don’t listen to this just now*…*…they don’t think it is particularly important or interesting I would like to find other ways to meet …perhaps it’s not the right time.*^40^^(p.4)^

They also addressed differences between pediatric and adult health care.

*The paediatric model is quite different from the adult model of care in that if they don’t come to clinic…we chase them, whereas in the adult field…it is up to them to do the chasing…the adult model is…here is the information, do with it what you will. In the pediatric scene…we don’t…give up on them…they’re babied you know. (Pediatrician)*^51^^(p.766)^

##### Category 5.2: HCPs’ suggestions to improve transition

This category was derived from 13 findings identified in 4 studies.^39^,^40^,^53^,^55^ HCPs mentioned many suggestions to improve transition that included more structured transition, education of young adults, and coordinating activities between pediatric and adult health care mainly by a key person or a nurse working in both sites.

*I think it’s good, now we get a totally different offer from the PDC [pediatric diabetes clinic] thanks to the new nurse from pediatrics, it gives us so much more, there are so many things that we don’t always think about on the adult side and vice versa, we have already understood that the nurse has implemented certain things that no one thought about before. (Adult HCP)*^40^^(p.6)^

## Discussion

This qualitative systematic review has provided a comprehensive overview of the experiences and perspectives of adolescents with T1D, their parents, and HCPs regarding the transition to adult health care. The 25 studies included in the review resulted in 154 unequivocal and 6 credible findings that were grouped into 12 categories. Five synthesized findings were identified; the first 3 synthesized findings focused on the perspectives of adolescents and young adults with T1D, while the fourth and fifth synthesized findings focused on parents and HCPs perspectives, respectively.

### Perceptions of transition

The first synthesized finding explores how adolescents and young adults perceive transition to adult health care. Generally, the adolescents and young adults appreciated the transition as a forward step in their developmental process and were ready to take responsibility over their diabetes management. However, they mostly felt inadequately prepared, and they needed continued support and education to acquire the knowledge and skills to be independent and autonomous in adult health care. Similar findings are described in previous qualitative reviews exploring the transition experience of adolescents with different chronic diseases, where they reported a spectrum of feelings regarding the transition to adult care.^60^,^61^ This dichotomy in experiences suggests a critical need for customized transition programs that address individual readiness levels. Programs that incorporate self-management education and foster autonomy may enhance the confidence of those who feel unprepared, ultimately improving their transition experience. The ongoing process of assessment of transition readiness and self-management skills provides the opportunity to develop targeted interventions and educational materials based on identified deficits and individual needs, and this can help to achieve successful transition.^62^-^64^

### Challenges for transition

The second synthesized finding comprises 2 categories relating to perceived challenges for transition, as voiced by adolescents and young adults. The first category reports difficulties in navigating changes in health care systems. Many adolescents and young adults described differences between adult and pediatric health care settings. They described pediatric care as a safe, comfortable environment with more frequent follow-up and better multidisciplinary care, while the adult care is less organized with less personal support, less frequent follow-ups, separation of services, and shorter consultations that could not address all their needs. They also reported significant difficulties navigating the complexities of the adult health care system, including long waiting times, misinformation, and an overwhelming bureaucratic process.

The second category of the second finding relates to the burden of managing T1D, compounded by competing life responsibilities such as college and work, which often exacerbate feelings of anxiety and frustration. The emotional weight of living with a chronic condition can lead to avoidance behaviors, further complicating effective self-management. These negative feelings associated with diabetes are described in literature as diabetes distress,^65^ and there is quantitative evidence that it is associated with lower transition readiness.^66^-^68^

The findings illustrate a complex dynamic in the transition process, where adolescents grapple with the challenge of shifting responsibility from caregivers to themselves. Many young individuals expressed difficulty in gaining the autonomy necessary for effective self-management of their diabetes, often citing that their caregivers did not provide adequate space for independence. This is particularly concerning, as fostering autonomy is essential for developing the skills required for managing their medical care.^38^,^46^

Simultaneously, parents described a significant transformation in their roles from active diabetes managers to more supportive and advisory figures. Many parents acknowledged their struggle to relinquish control, even while recognizing the importance of this transition for their child’s growth. This tension highlights the need for targeted strategies that work for both parents and adolescents to facilitate a gradual shift of diabetes management responsibility from shared management by the adolescent and parents to more independent management by the adolescents. This will help ensure that young individuals are equipped with the necessary skills and confidence to navigate their health management effectively. These insights highlight the importance of addressing both systemic and personal barriers in transition planning.

### Transition facilitators

The third synthesized finding looks at the successful transition from pediatric to adult care for adolescents with T1D as a multifaceted process. The shared facilitators identified across the perspectives of adolescents, parents, and HCPs emphasize the importance of preparation, support networks, and systemic improvements.

A prominent finding in our synthesis highlights the importance of preparation through education and fostering self-management skills. Adolescents expressed a strong desire for enhanced preparation as they transition to adult care. This finding is congruent with quantitative evidence showing that structured educational interventions that promote independence can significantly impact the success of the transition.^69^-^71^

Moreover, the significance of social support was highly emphasized. Adolescents and parents alike recognized the value of connecting with peers who share similar experiences. This peer support can help mitigate feelings of isolation and provide practical strategies for managing diabetes in adulthood. Initiatives that promote peer mentorship and facilitate connections among young adults with T1D could enhance emotional resilience and self-efficacy. This is congruent with quantitative evidence, where participating in support groups was associated with improvement in glycemic control, diabetes-related self-care behaviors, and a decrease in diabetes distress.^72^,^73^

Coordination initiatives were suggested by adolescents, parents, and HCPs. The presence of a transition coordinator or a designated point of contact was widely considered an important contributor to successful transition and ensures continuity of care. These findings are congruent with quantitative evidence showing that having a transition coordinator as part of a comprehensive transition program can lead to favorable transition outcomes.^26^,^74^ Furthermore, this aligns with National Institute for Health and Care Excellence (NICE) guidelines for the transition of adolescents to adult health care that recommend the presence of a named worker to support adolescents during transition, ideally someone with whom the young person has a meaningful relationship (eg, nurse from pediatric clinic). In addition to being a primary contact person for the patient, the transition coordinator should help the adolescent to engage with the process. They should also give advice, information, and support, and link the patient to the various practitioners involved in their support, including the named general practitioner.^75^

Adolescents and young adults also identified a positive relationship with adult HCPs, who promoted autonomy and shared decision-making as a key facilitator of their transition to adult care. This finding is consistent with literature suggesting that a strong patient-provider relationship has a positive impact on diabetes self-care and glycemic control in adults.^76^-^78^ Hence, focusing on building robust rapport between transition patients and adult providers may not only enhance the transition experience, but also have a long-term positive impact on glycemic control.

### Parental and health care practitioners’ perspectives

The fourth synthesized finding relates to parental experience of transition. The transition process is not a concern for the adolescents only; parents also navigated significant changes as their roles shifted from primary caregivers to supportive advisers. This struggle to “let go” can create tension and uncertainty, highlighting the need for educational resources and support networks specifically designed for parents.^41^ Addressing these challenges through targeted interventions can empower parents and enhance their confidence in allowing their children to take charge of their diabetes management.^41^,^52^ This finding is congruent with previous systematic reviews exploring the experiences of parents of adolescents and young adults with different chronic conditions, where parents expressed consistent concerns about their children’s transition.^79^,^80^

In the fifth synthesized finding, HCPs expressed uncertainty about which of the available transition strategies to recommend, reflecting a broader systemic issue of lack of structured protocols. This aligns with a previous survey of pediatric endocrinologists, where the majority reported a lack of structured transition and a lack of transition training as barriers to transition.^81^ Their suggestions for improvement, focusing on coordination and structured education, indicate a consensus on the need for systemic change. This highlights an opportunity for health care systems to develop comprehensive transition frameworks that not only support young adults but also engage their families and health care teams in a collaborative approach.

### Strengths and limitations of the review

This systematic review included the perspectives of adolescents and young adults with T1D, their parents, and HCPs, thereby ensuring a comprehensive understanding of the transition experience beyond the scope of a quantitative review, which is crucial for the successful design of feasible and effective health care transition interventions for T1D.

This systematic review adhered to the robust systematic methods as outlined by JBI,^34^ and was reported according to PRISMA guidelines.^32^

The methodological quality of most of the included studies was high. Additionally, as reported in the Summary of Findings, the ConQual scores of the synthesized findings were moderate, thereby demonstrating that the findings from this review are robust and can be used for forming the basis of recommendations.

The systematic review identified and included studies from a variety of countries, but mainly from North America, Europe, and Australia, which limited our ability to explore any potential cultural issues. Studies from other health care systems would have given further knowledge of cultural similarities and differences.

## Conclusion

The transition from pediatric to adult health care for adolescents with T1D is a complex process that requires careful planning and support. This qualitative systematic review highlights the varied perceptions of adolescents, ranging from confidence to anxiety, and identifies significant barriers within health care systems and the challenges of living with T1D.

Adolescents and their families require comprehensive support systems that address emotional and practical aspects of transition. HCPs play a critical role in this process, and there is an urgent need for structured transition programs that foster independence while ensuring continuity of care. Additionally, the perspectives of parents and HCPs must be integrated into transition strategies to enhance the overall effectiveness of these programs.

By implementing the outlined recommendations, we can create a more supportive and effective transition pathway for adolescents with T1D, ultimately leading to improved health outcomes and quality of life in their adult years. The engagement of all stakeholders, including adolescents, parents, HCPs, and policymakers, is essential to drive meaningful change in the transition process for young individuals managing T1D.

### Recommendation for practice and policy

- Policies that promote coordinated care between pediatric and adult health care systems are recommended. This may include shared protocols, communication strategies, and a designated transition coordinator. (Grade A)
- Interdisciplinary collaboration between HCPs from pediatric and adult settings is recommended during the transition period to ensure comprehensive support and continuity of care. An interdisciplinary collaborative care model may include joint appointments, shared care models, or a transition coordinator. (Grade A)
- Peer support activities and mentoring programs can be suggested for providing guidance and emotional support to adolescents and young adults during the transition. (Grade B)
- Supporting strategies for parents can be suggested to address their needs during the transition and to assist them in adapting to their evolving role. (Grade B)

### Recommendation for future research

- More qualitative research should be conducted in underrepresented countries, especially in Africa and Asia, and other diverse demographic groups (eg, ethnic minorities, low-income families) to identify specific barriers and facilitators that may vary across populations.
- Research should focus on parental experience and the challenges they face during the transition of their children, and assess how parents’ needs can be supported during this crucial stage. Also, more attention to HCPs’ perspectives could help to explore challenges in more depth and provide suggestions to improve the transition services.

## Funding

MGA is the recipient of funding from the Egyptian Ministry of High Education. The funder had no involvement in the conduct of the review.

## Author contributions

MGA: Designed the review; developed the search strategy; and conducted the search, screening of the studies, critical appraisal of the included studies with JLB, data extraction with FP, analysis of findings, and drafting the initial manuscript. EW: Designed the review, conducted initial screening with MGA, oversaw of analysis of findings to develop synthesized findings, and reviewed the manuscript. HB: Designed the review, resolved any disagreement regarding including/excluding studies, oversaw analysis of findings to develop synthesized findings, and reviewed the manuscript. FP: Conducted data extraction with MGA. JL: Critically revised the manuscript. JLB: Designed the review, revised the search strategy, provided overall methodological supervision, acted as second reviewer of critical appraisal of included papers, contributed in ConQual ranking of included studies, and critically revised the manuscript.

## Appendix I: Search strategy

**Table:**

MEDLINE (Ovid)		
Search conducted: May 28, 2025		
#	Search	Records retrieved
1	“diabetes mellitus type 1” or “type 1 diabetes mellitus” or “type 1 diabetes” or “diabetes type 1” or “juvenile diabetes” or “insulin dependent diabetes” or T1D or IDDM or diabet*	
2	“Transition to Adult Care” or “transition to adult health care”.mp.	
3	transition* adj6 (care or unit or clinic or service or center or centre or intervention* or program or model)	
4	2 OR 3	
5	1 AND 4	1102

**Table:**

Embase (Ovid)		
Search conducted: May 28, 2025		
#	Search	Records retrieved
1	“diabetes mellitus type 1” or “type 1 diabetes mellitus” or “type 1 diabetes” or “diabetes type 1” or “juvenile diabetes” or “insulin dependent diabetes” or T1D or IDDM or diabet*	
2	“Transition to Adult Care” or “transition to adult health care”.mp.	
3	transition* adj6 (care or unit or clinic or service or center or centre or intervention* or program or model)	
4	2 OR 3	
5	1 AND 4	2831

**Table:**

PsycINFO (Ovid)		
Search conducted: May 28, 2025		
#	Search	Records retrieved
1	“diabetes mellitus type 1” or “type 1 diabetes mellitus” or “type 1 diabetes” or “diabetes type 1” or “juvenile diabetes” or “insulin dependent diabetes” or T1D or IDDM or diabet*	
2	“Transition to Adult Care” or “transition to adult health care”.mp.	
3	transition* adj6 (care or unit or clinic or service or center or centre or intervention* or program or model)	
4	2 OR 3	
5	1 AND 4	162

**Table:**

CINAHL (EBSCOhost)	
Search conducted: May 28, 2025	
Search	Records retrieved
(("transition to adult care” or “transition to adult health care”) OR (transition* N6 (care or unit or clinic or service or center or centre or intervention* or program or model))) AND ("diabetes mellitus type 1” or “type 1 diabetes mellitus” or “type 1 diabetes” or “diabetes type 1” or “juvenile diabetes” or “insulin dependent diabetes” or T1D or IDDM or diabet*)	853

**Table:**

PubMed	
Search conducted: May 28, 2025	
Search	Records retrieved
("transition to adult care"[tiab] OR “transition to adult health care"[tiab] OR (transition*[tiab] AND (care[tiab] OR unit[tiab] OR clinic[tiab] OR service[tiab] OR center[tiab] OR centre[tiab] OR intervention*[tiab] OR program[tiab] OR model[tiab]))) AND ("diabetes mellitus type 1"[tiab] OR “type 1 diabetes mellitus"[tiab] OR “type 1 diabetes"[tiab] OR “diabetes type 1"[tiab] OR “juvenile diabetes"[tiab] OR “insulin dependent diabetes"[tiab] OR T1D[tiab] OR IDDM[tiab] OR diabet*[Mesh])	1245

**Table:**

Cochrane Library	
Search conducted: May 28, 2025	
Search	Records retrieved
“Transition to Adult Care” OR “transition to adult health care” OR “transition” adj6 (care or unit or clinic or service or center or centre or intervention* or program or model AND “diabetes mellitus type 1” or “type 1 diabetes mellitus” or “type 1 diabetes” or “diabetes type 1” or “juvenile diabetes” or “insulin dependent diabetes” or T1D or IDDM or diabet*	110

## Appendix II: Studies ineligible following full-text review

1. Agarwal S, Garvey KC, Raymond JK, Schutta MH. Perspectives on care for young adults with type 1 diabetes transitioning from pediatric to adult health systems: a national survey of pediatric endocrinologists. Pediatr Diabetes 2016;18(7):524.
  *Reason for exclusion:* ineligible study design
2. Allen D, Gregory J. The transition from children’s to adult diabetes services: understanding the ‘problem.’ Diabet Med 2009;26(2):162-6.
  *Reason for exclusion:* ineligible study design
3. Babler E, Strickland CJ. Moving the Journey towards independence: adolescents transitioning to successful diabetes self-management. J Pediatr Nurs 2015;30(5):648-60.
  *Reason for exclusion:* ineligible phenomena of interest
4. Babler E, Strickland CJ. Normalizing: adolescent experiences living with type 1 diabetes. Diabetes Educ 2015;41(3):351-60.
  *Reason for exclusion:* ineligible phenomena of interest
5. Begley T. Transition to adult care for young people with long-term conditions. Br J Nurs 2013;22(9):506-10.
  *Reason for exclusion:* ineligible participants
6. Bomba F, Herrmann-Garitz C, Schmidt J, Schmidt S, Thyen U. An assessment of the experiences and needs of adolescents with chronic conditions in transitional care: a qualitative study to develop a patient education programme. Health Soc Care Community 2017;25(2):652-66.
  *Reason for exclusion:* ineligible participants
7. Bridgett M, Abrahamson G, Ho J. Transition, It’s more than just an event: supporting young people with type 1 diabetes. J Pediatr Nurs 2015;30(5):e11-4.
  *Reason for exclusion:* ineligible study design
8. Burden JM-S. Parent and adolescent readiness in the transition to adolescent diabetic self care [dissertation]. Michigan State University; 2010.
  *Reason for exclusion:* ineligible phenomena of interest
9. Busse-Voigt FP, Kiess W, Stumvoll M, Hering N, Kapellen TM. [Building bridges during transition of patients with type 1 diabetes. Adult medical and pediatric point of views]. Internist (Berl) 2009;50(10):1194. [German]
  *Reason for exclusion:* ineligible study design
10. Cassidy CE, Kontak JC, Pidduck J, Higgins A, Anderson S, Best S, *et al.* Provider perspectives of barriers and facilitators to the transition from pediatric to adult care: a qualitative descriptive study using the COM-B model of behaviour. J Transit Med 2022;4(1):20220003.
  *Reason for exclusion:* ineligible participants
11. Castensøe-Seidenfaden P, Teilmann G, Kensing F, Hommel E, Olsen BS, Husted GR. Isolated thoughts and feelings and unsolved concerns: adolescents’ and parents’ perspectives on living with type 1 diabetes – a qualitative study using visual storytelling. J Clin Nurs 2017;26(19–20):3018-30.
  *Reason for exclusion:* ineligible phenomena of interest
12. Chiang Y-T, Yu H-Y, Lo F-S, Chen C-W, Huang T-T, Chang C-W, *et al.* Emergence of a butterfly: the life experiences of type 1 diabetes Taiwanese patients during the 16-25 years old transition period. Int J Qual Stud Health Well-being 2020;15(1):1748362.
  *Reason for exclusion:* ineligible phenomena of interest (general transition to adulthood)
13. Colver A, Pearse R, Watson RM, Fay M, Rapley T, Mann KD, *et al.* How well do services for young people with long term conditions deliver features proposed to improve transition? BMC Health Serv Res 2018;18(1):1-10.
  *Reason for exclusion:* ineligible participants
14. Court JM. Issues of transition to adult care. J Paediatr Child Health 1993;29 Suppl 1:S53-5.
  *Reason for exclusion:* ineligible study design
15. Court JM. Outpatient-based transition services for youth. Pediatrician 1991;18(2):150-6.
  *Reason for exclusion:* ineligible study design
16. Coyne I, Sheehan A, Heery E, While A. Health care transition for adolescents and young adults with long-term conditions: qualitative study of patients, parents and healthcare professionals’ experiences in Ireland. Arch Dis Child 2019;104(Supplement 3):A83.
  *Reason for exclusion:* ineligible participants
17. Crosnier H, Tubiana-Rufi N. [Modalities of the transition of diabetic adolescents from pediatrics to adult structures in the Paris-Île-de-France region: a call for collaborative work to improve the quality of care]. Arch Pédiatrie 1998;5(12):1327-33. [French]
  *Reason for exclusion:* ineligible study design
18. de Beaufort C, Jarosz-Chobot P, Frank M, de Bart J, Deja G. Transition from pediatric to adult diabetes care: smooth or slippery? Pediatr Diabetes 2010;11(1):24-7.
  *Reason for exclusion:* ineligible study design
19. Daneman D, Nakhla M. Moving on: transition of teens with type 1 diabetes to adult care. Diabetes Spectr 2011;24(1):14-8.
  *Reason for exclusion:* ineligible study design
20. Datz N, Kordonouri O, Danne T. [When people with type 1 diabetes become adults - Diabetes technology and transition - do we need new models?]. Dtsch Med Wochenschr 2021;146(18):1200-5. [German]
  *Reason for exclusion:* ineligible study design
21. Dimitropoulos G, Morgan-Maver E, Allemang B, Schraeder K, Scott SD, Pinzon J, *et al.* Health care stakeholder perspectives regarding the role of a patient navigator during transition to adult care. BMC Health Serv Res 2019;19(1):1-10.
  *Reason for exclusion:* ineligible participants
22. Dovey-Pearce G, Hurrell R, May C, Walker C, Doherty Y. Young adults’ (16-25 years) suggestions for providing developmentally appropriate diabetes services: a qualitative study. Heal Soc Care Community 2005;13(5):409-19.
  *Reason for exclusion:* ineligible phenomena of interest
23. D’Sa S, Foley DJ, Hennigan K, Kelly-Conroy M, Quinn A, Norris M, *et al.* Exploring the attitudes and experiences of adolescents with type 1 diabetes towards transition of care. J Public Heal 2023;31(7):1151-6.
  *Reason for exclusion:* ineligible study design
24. Duke DC, Raymond JK, Shimomaeda L, Harris MA. Recommendations for transition from pediatric to adult diabetes care: patients’ perspectives. Diabetes Manag 2013;3(4):297-304.
  *Reason for exclusion:* ineligible study design
25. Eiser C, Flynn M, Green E, Havermans T, Kirby R, Sandeman D, *et al.* Coming of age with diabetes: patients’ views of a clinic for under-25 year olds. Diabet Med 1993;10(3):285-9.
  *Reason for exclusion:* ineligible study design
26. Ersig AL, Tsalikian E, Coffey J, Williams JK. Stressors in Teens with type 1 diabetes and their parents: immediate and long-term implications for transition to self-management. J Pediatr Nurs 2016;31(4):390-6.
  *Reason for exclusion:* ineligible phenomena of interest
27. Flor M V, Morato MJI, Vergara CY, Cardona-Hernandez R, Alvarez MG, Donlo IC, *et al.* Type 1 diabetes patient experiences before and after transfer from a paediatric to an adult hospital. Patient Prefer Adherence 2022;16:2229-46.
  *Reason for exclusion:* ineligible study design
28. Garvey KC, Foster NC, Agarwal S, DiMeglio LA, Anderson BJ, Corathers SD, *et al.* Health care transition preparation and experiences in a U.S. national sample of young adults with type 1 diabetes. Diabetes Care 2017;40(3):317-24.
  *Reason for exclusion:* ineligible study design
29. Garvey K, Wolpert H, Laffel L, Rhodes E, Wolfsdorf J, Finkelstein J. Health care transition in young adults with type 1 diabetes: barriers to timely establishment of adult diabetes care. Endocr Pract 2013;19(6):946-52.
  *Reason for exclusion:* ineligible study design
30. Garvey KC, Wolpert HA, Rhodes ET, Laffel LM, Kleinman K, Beste MG, *et al.* Health care transition in patients with type 1 diabetes: young adult experiences and relationship to glycemic control. Diabetes Care 2012;35(8):1716-22.
  *Reason for exclusion:* ineligible study design
31. Goethals ER, La Banca RO, Forbes PW, Telo GH, Laffel LM, Garvey KC. Health care transition in type 1 diabetes: perspectives of diabetes care and education specialists caring for young adults. Diabetes Educ 2020;46(3):252-60.
  *Reason for exclusion:* ineligible study design
32. Hauschild M, Elowe-Gruau E, Dwyer A, Aquarone M-P, Unal S, Jornayvaz FR, *et al.* [Transition in diabetology]. Rev Med Suisse 2015;11(462):450-5. [French]
  *Reason for exclusion:* ineligible study design
33. Helgeson VS, Reynolds KA, Snyder PR, Palladino DK, Becker DJ, Siminerio L, *et al.* Characterizing the transition from paediatric to adult care among emerging adults with Type 1 diabetes. Diabet Med 2013;30(5):610-5.
  *Reason for exclusion:* ineligible study design
34. Holtz BE, Mitchell KM, Holmstrom AJ, Cotten SR, Hershey DD, Dunneback JK, *et al.* Teen and parental perspectives regarding transition of care in type 1 diabetes. Child Youth Serv Rev 2020;110(January):104800.
  *Reason for exclusion:* ineligible phenomena of interest
35. Huang JS, Gottschalk M, Pian M, Dillon L, Barajas D, Bartholomew LK. Transition to adult care: systematic assessment of adolescents with chronic illnesses and their medical teams. J Pediatr 2011;159(6):994-998.e2.
  *Reason for exclusion:* ineligible participants
36. Jackson CC, Albanese-O’Neill A. Supporting the student’s graduated independence in diabetes care. NASN Sch Nurse 2016;31(4):202-4.
  *Reason for exclusion:* ineligible study design
37. Kichler JC, Gyemi A, Papak R, Tapp K, Grandi B, Lucier K. “I’ll just forever be that person who stands in the middle of the dance floor drinking a juice box”: supporting the transition to adulthood with type 1 diabetes in a post-secondary university/college setting. Diabetes Spectr 2023;36(4):354-63.
  *Reason for exclusion:* ineligible phenomena of interest
38. Kipps S, Bahu T, Ong K, Ackland FM, Brown RS, Fox CT, *et al.* Current methods of transfer of young people with Type 1 diabetes to adult services. Diabet Med 2002;19(8):649-54.
  *Reason for exclusion:* ineligible study design
39. Klein K, Wheeler M, Yonkaitis CF. College-bound: transition planning strategies for students with type 1 diabetes. NASN Sch Nurse 2019;34(1):17-20.
  *Reason for exclusion:* ineligible phenomena of interest (transition to college)
40. Lee YA. Diabetes care for emerging adults: transition from pediatric to adult diabetes care systems. Ann Pediatr Endocrinol Metab 2013;18(3):106.
  *Reason for exclusion:* ineligible study design
41. Lee J. Diagnosis and management of pediatric type 1 diabetes mellitus. J Korean Med Assoc 2021;64(6):425-31.
  *Reason for exclusion:* ineligible study design
42. Lundin CS, Öhrn I, Danielson E. From multidimensional support to decreasing visibility: a field study on care culture in paediatric and adult diabetes outpatient clinics. Int J Nurs Stud 2008;45(2):180-90.
  *Reason for exclusion:* ineligible study design
43. Lyons SK, Helgeson VS, Witchel SF, Becker DJ, Korytkowski MT. Physicians’ self-perceptions of care for emerging adults with type 1 diabetes. Endocr Pract 2015;21(8):903-9.
  *Reason for exclusion:* ineligible study design
44. Malivoir S, Gueniche K. “Child-adult” transition - adolescence: when illness appears…. Endocr Dev. 2018;33:10-6.
  *Reason for exclusion:* ineligible study design
45. Marsh KK, Bush RA, Connelly CD. Exploring perceptions and use of the patient portal by young adults with type 1 diabetes: a qualitative study. Health Informatics J 2020;26(4):2586-96.
  *Reason for exclusion:* ineligible phenomena of interest
46. McGill M. How do we organize smooth, effective transfer from paediatric to adult diabetes care? Horm Res 2002;57 Suppl 1(SUPPL. 1):66-8.
  *Reason for exclusion:* ineligible study design
47. Meadows AL, Marsac ML. Early-life trauma and diabetes management: an under-recognized phenomenon in transition-aged youth. Clin Diabetes 2020;38(1):93.
  *Reason for exclusion:* ineligible study design
48. Mellerio H, Dumas A, Alberti C, Guilmin-Crépon S, Gastaldi M, Loïc Passini, *et al.* Are transition preparation consultations for adolescents with chronic conditions valuable? A mixed-methods study. Eur J Pediatr 2022;18(7):2849-61.
  *Reason for exclusion:* ineligible participants
49. Menon J, Peter AM, Nayar L, Kannankulangara A. Need and feasibility of a transition clinic for adolescents with chronic illness: a qualitative study. Indian J Pediatr 2020;87(6):421-6.
  *Reason for exclusion:* ineligible participants
50. Modrzyńska K, Szadkowska A. [Transition of young adults with type 1 diabetes mellitus from a pediatric to adult diabetes clinic - problems, challenges and recommendations]. Pediatr Endocrinol Diabetes Metab 2015;20(3):116-22. [Polish]
  *Reason for exclusion:* ineligible study design
51. Morsa M, Lombrail P, Boudailliez B, Godot C, Jeantils V, Gagnayre R. A qualitative study on the educational needs of young people with chronic conditions transitioning from pediatric to adult care. Patient Prefer Adherence 2018;12:2649-60.
  *Reason for exclusion:* ineligible participants
52. Nasrabadi AN, Dehkordi LM. Diabetes experiences of transition from childhood to adulthood care. Florence Nightingale J Nurs 2022;30(1):3-8.
  *Reason for exclusion:* ineligible phenomena of interest
53. Ng AH, Pedersen ML, Rasmussen B, Rothmann MJ. Needs of young adults with type 1 diabetes during life transitions - an Australian-Danish experience. Patient Educ Couns 2022;105(5):1338-41.
  *Reason for exclusion:* ineligible phenomena of interest
54. Parfitt G. Improving the young person’s experience of transition: lessons from Wales. Paediatr Care 2008;20(9):27-30.
  *Reason for exclusion:* ineligible study design
55. Peeters MAC, de Haan HG, Bal RA, van Staa AL, Sattoe JNT. Active involvement of young people with T1DM during outpatient hospital consultations: opportunities and challenges in transitional care services. Patient Educ Couns 2022;105(6):1510-7.
  *Reason for exclusion:* ineligible phenomena of interest
56. Perry L, Dunbabin J, Xu X, Lowe J, Acharya S, James S, *et al.* Service use of young people with Type 1 diabetes after transition from paediatric to adult-based diabetes health care. Aust Health Rev 2020;44(4):601-8.
  *Reason for exclusion:* ineligible study design
57. Ramchandani N, Way N, Melkus GD, Sullivan-Bolyai S. Challenges to diabetes self-management in emerging adults with type 1 diabetes. Diabetes Educ 2019;45(5):484-97.
  *Reason for exclusion:* ineligible phenomena of interest
58. Price CS, Corbett S, Lewis-Barned N, Morgan J, Oliver LE, Dovey-Pearce G. Implementing a transition pathway in diabetes: a qualitative study of the experiences and suggestions of young people with diabetes. Child Care Health Dev 2011;37(6):852-60.
  Reason for exclusion: ineligible phenomena of interest
59. Rasmussen B, Ward G, Jenkins A, King SJ, Dunning T. Young adults’ management of type 1 diabetes during life transitions. J Clin Nurs 2011;20(13-14):1981-92.
  *Reason for exclusion:* ineligible phenomena of interest
60. Raymond JK, Duke DC, Shimomaeda L, Harris MA. Looking forward to transition: perspectives on transition from pediatric to adult diabetes care. Diabetes Manag (Lond) 2013;3(4):10.2217/dmt.13.27.
  *Reason for exclusion:* ineligible study design
61. Reiss JG, Gibson RW, Walker LR. Health care transition: youth, family, and provider perspectives. Pediatrics 2005;115(1):112-20.
  *Reason for exclusion:* ineligible participants
62. Sawyer B, Hilliard E, Hackney KJ, Stastny S. Barriers and strategies for type 1 diabetes management among emerging adults: a qualitative study. Clin Med Insights Endocrinol Diabetes 2022;15.
  *Reason for exclusion:* ineligible phenomena of interest
63. Schulman R, Chafe R, Guttmann A. Transition to adult diabetes care: description of practice in the Ontario Pediatric Diabetes Network. Can J Diabetes 2019;43(4):283-9.
  *Reason for exclusion:* ineligible study design
64. Schlüter S, Lange K. From the perspective of diabetes practice: young adults with type 1 diabetes. Diabetologe 2022;18(2):114-21.
  *Reason for exclusion:* ineligible study design
65. Skedgell KK, Cao VT, Gallagher KA, Anderson BJ, Hilliard ME. Defining features of diabetes resilience in emerging adults with type 1 diabetes. Pediatr Diabetes 2021;22(2):345-53.
  *Reason for exclusion:* ineligible phenomena of interest
66. Skolness VA. Success through transition: a transition planning checklist for diabetes care transition [dissertation]. North Dakota State University; 2015.
  *Reason for exclusion:* ineligible study design
67. Sparud-Lundin C, Öhrn I, Danielson E. Redefining relationships and identity in young adults with type 1 diabetes. J Adv Nurs 2010;66(1):128-38.
  *Reason for exclusion:* ineligible phenomena of interest
68. Sullivan-Bolyai S, Bova C, Johnson K, Cullen K, Jaffarian C, Quinn D, *et al.* Engaging teens and parents in collaborative practice: perspectives on diabetes self-management. Diabetes Educ 2014;40(2):178-90.
  *Reason for exclusion:* ineligible phenomena of interest
69. van Staa AL, Jedeloo S, van Meeteren J, Latour JM. Crossing the transition chasm: experiences and recommendations for improving transitional care of young adults, parents and providers. Child Care Health Dev 2011;37(6):821-32.
  *Reason for exclusion:* ineligible participants
70. Whittemore R, Zincavage RM, Jaser SS, Grey M, Coleman JL, Collett D, *et al.* Development of an eHealth program for parents of adolescents with type 1 diabetes. Diabetes Educ 2018;44(1):72-82.
  *Reason for exclusion:* ineligible phenomena of interest
71. Wilson V. Students’ experiences of managing type 1 diabetes. Paediatr Nurs 2010;22(10):25-8.
  *Reason for exclusion:* ineligible phenomena of interest
72. White KN. Transition experiences of the chronically ill adolescent. Florida Atlantic University; 2014.
  *Reason for exclusion:* ineligible participants
73. Yi-Frazier J, Senturia K, Wright D, Lind C, Malik F. The clock is ticking: Parental stress around emerging adulthood for adolescents with type 1 diabetes. Pediatr Diabetes 2021;22(SUPPL 29):12-3.
  *Reason for exclusion:* ineligible phenomena of interest
74. Fernandes SM, O’Sullivan-Oliveira J, Landzberg MJ, Khairy P, Melvin P, Sawicki GS, *et al.* Transitioning and transfer of adolescents and young adults with pediatric onset chronic disease: the patient and parent perspective. J Pediatr Rehabil Med 2014;7(1):43.
  *Reason for exclusion:* ineligible participants
75. Rasmussen B, O’Connell B, Dunning P, Cox H. Young women with type 1 diabetes’ management of turning points and transitions. Qual Health Res 2007;17(3):300-10.
  *Reason for exclusion:* ineligible phenomena of interest
76. Chafe R, Shulman R, Guttmann A, Aubrey-Bassler K. Adolescent patients with chronic health conditions transitioning into adult care: what role should family physicians play? Can Fam Physician 2019;65(5):317.
  *Reason for exclusion:* ineligible study design
77. Turns M. Young people with type 1 diabetes and their transition to adult services. Br J Community Nurs 2013;18(1).
  *Reason for exclusion:* ineligible study design

## Appendix III: Characteristics of included studies

**Table:**

Study, year, country	Model for data collection and analysis	Phenomena of interest	Setting/context/culture	Participant characteristics/sample size	Author conclusions
Allen *et al.*,^52^ 2011, UK	Longitudinal qualitative case studies: Interviews were undertaken at 3 time points over 12–18 months	The experiences of young people and their carers during the transition from child to adult diabetes services	5 different services in the UK	Potential cases were identified by appropriately placed service providers and a purposive sample selected.	There is a clear need to develop service structures that recognize the continuing role played by mothers in the diabetes care of young adults.
	Theoretical framework: Strauss and co-workers’ concept of an illness trajectory was used to frame the study		Face-to-face interviews were carried out mainly in the young person’s home, with a minority undertaken in alternative venues such as cafes.	Health service 1: 5 young people (4 males, 1 female), mean age 15.2 years, mean diabetes duration 6.8 years	
				Number of mothers interviewed: 5	
				Health service 2: 7 young people (5 males, 2 females), mean age 16.8 years, mean diabetes duration 10.2 years	
				Number of mothers interviewed: 6	
				Health service 3: 9 young people (5 males, 4 females), mean age 17.6 years, mean diabetes duration 10.2 years	
				Number of mothers interviewed: 7	
				Health service 4: 11 young people (3 males, 8 females), mean age 14.7 years, mean diabetes duration 4.5 years	
				Number of mothers interviewed: 10	
				Health service 5: 14 young people (7 males, 7 females), mean age 17 years, mean diabetes duration 9 years	
				Number of mothers interviewed 11	
Butalia *et al.*,^47^ 2020, Canada	Focus groups of youth with type 1 diabetes in transition from pediatric to adult diabetes care and their parents in Calgary, Alberta, between June and August 2014	To gain insight into how to improve the transition of youth with type 1 diabetes from pediatric to adult diabetes care from the patients’ and parents’ perspective	This study was conducted in the Alberta Children’s Hospital in Calgary, Alberta, a large urban city.	Group 1: 7 youth (3 males, 4 females) with type 1 diabetes who were pre-transfer (median age 17.5 years, interquartile range 1.3 years)	This study describes specific areas that may improve diabetes transfer and transition from pediatric to adult diabetes care. This information can help inform clinical care delivery for transition and the development of programs, strategies, and interventions to improve transition care.
	Thematic analysis was used for data analysis		The Alberta Children’s Hospital diabetes program and the adult diabetes care program provide comprehensive, multidisciplinary diabetes care; however, at the time of the focus groups, the transition process included only a brief educational program delivered during regular pediatric clinical care visits, and no transition coordinator was in place. The transition process started at age 14 as per the national transition guidelines. An electronic transition checklist was used by all the pediatric clinics to ensure transition topics were covered prior to transfer. Transfer from pediatric to adult care services occurs at 17 to 18 years of age at the center.	Group 2: 4 young adults (2 males, 2 females) with type 1 diabetes who were post-transfer (median age 23.5 years, interquartile range 1.2 years)	
				Group 3: 3 parents of youth with type 1 diabetes who had transferred from pediatric to adult care	
Garvey *et al.*,^42^ 2014, USA	5 focus groups (stratified by current level of glycemic control [A1C]) were conducted	Experiences of transition preparation and care transfer as voiced by post-transition emerging adults with T1D	A comprehensive diabetes center in the northeastern United States	5 x 75-minute focus groups	Findings identify modifiable deficiencies in the T1D transition process and underscore the importance of a planned transition with enhanced preparation by pediatric clinics as well as developmentally tailored patient orientation in the adult clinic setting.
	Data were analyzed using thematic analysis		Although the diabetes center has both pediatric and adult units, there is no established structured transition of care program.	Each group consisted of 3-7 patients (n=26, mean age 26.2 ± 2.5 years, diabetes duration 16.3 ± 4.7 years, mean age at transition from pediatric to adult care 20.3 ± 3.2 years, 62% female, 85% with college degree, 81% non-Hispanic White).	
				Groups were formed based on participants’ current level of glycemic control (A1C): lower A1C ≤8.5% and higher A1C >8.5%. Three groups had lower A1C values (mean for lower A1C groups = 7.4%± 0.6%), and 2 groups had higher A1C values (mean = 9.8% ± 1.0%).	
				Purposive sampling strategies used to identify English-speaking emerging adults with T1D who were diagnosed ≤18 years old and had T1D for at least 5 years.	
Guitard-Munnich and Houdan,^57^ 2019, France	Semi-structured interviews were conducted	Patients’ perception of their care during transition from pediatric to adult medicine in order to identify areas of satisfaction and dissatisfaction as experienced and formulated by them	The participants were drawn from 2 cohorts: a longitudinal cohort recruited in pediatrics (n=61) and a cross-sectional cohort recruited after transfer to adult diabetology (n=70)	20 interviews were conducted with 11 patients recently treated in adult diabetology and 9 still treated in pediatrics.	This study highlights the importance of encouraging adolescents’ autonomy in the years leading up to transition. A key health care professional link between both services appears to facilitate smooth transition. Being flexible and supportive of both parents and adolescents, including the provision of mental health services, are other important considerations.
	Data were analyzed by framework analysis				
Hansen and Jensen,^58^ 2017, Denmark	Data were collected by semi-structured interviews in spring 2016	How adolescents with T1DM experienced the transition from pediatric to adult diabetes services with a focus on partnership and shared decision-making between the provider and the adolescent	The study was conducted 1 year after all adolescents (n=18) with T1DM transferred from the outpatient clinic at the Department of Pediatrics at Aarhus University Hospital to the outpatient clinic at the Department of Endocrinology and Internal Medicine at Aarhus University Hospital, Denmark.	Out of all adolescents (n=18) with T1DM who had transferred, 10 adolescents agreed to participate in the study [7 females, 3 males, mean age 17.2 years with mean age at diagnosis 8.7 years]	Findings highlight the importance of a specific relationship with the HCP and a planned and prepared transition. Being unaware of the transition process leads to a non-reflective transition and does not support the development of health literacy and partnership.
	Data analysis was an inductive process using systematic text condensation	The participants in the study went through the same cross-functional transition process over a year, starting in the fall 2014.			
Harris *et al.*,^39^ 2020, Australia	Semi-structured interviews were conducted during October and November 2016	Health care professionals’ experiences and perceptions of diabetes health care services, including during the period of transition	1 health jurisdiction in New South Wales, Australia	16 health care professionals	Findings identify opportunities for development in the planning and provision of specialist multidisciplinary health care support for this population. New ideas are needed for policy and practice innovation and for the infrastructure to facilitate this to ensure that young adults with T1D have access to consistent and coordinated diabetes health care services, particularly in nonmetropolitan settings.
	Data were analyzed using thematic analyses			Most were female (n=12, 75%) and registered nurses (n=12, 75%), working at differing levels of expertise and responsibility.	
				9 participants (56%) worked predominantly in metropolitan and 7 (44%) in regional/rural areas.	
				Recruitment was based on a snowball sampling technique, which began with members of an established diabetes service group.	
Hilliard *et al.*,^44^ 2014, USA	Semi-structured interviews conducted with the transition expectation group; the post-transition group completed a survey with open ended questions. Thematic synthesis used for analysis.	Concerns, expectations, preferences, and experiences of pre-transition adolescents and parents and post-transition young adults	For the transitions expectations group, adolescents aged 15–17 years with T1D were recruited during diabetes clinic visits at a pediatric tertiary medical center in the mid-Atlantic region of the United States.	The transition expectation group: 20 adolescents with T1D participated, all had at least 1 parent present (12 mothers, 9 fathers), and 2 interviews included 2 parents; mean participant age: 16.1 ± 0.6 years.	Standardizing transition preparation programs in pediatric care and introducing transition-oriented clinics for late adolescents and young adults prior to adult care may help address patients’ preferences and common transfer-related challenges.
			For the post-transition group, the diabetes educator from each of 3 participating pediatric diabetes clinics contacted young adults aged 18–22 years who were previously treated at their clinic to introduce the study and obtain informed consent.	Participants were 60% female, and all self-identified as Caucasian.	
				The mean A1C level was 8.7 ± 1.5%; mean age at diagnosis was 4.7 ± 3.1years.	
				In the post-transition group, 59 out of 70 eligible former patients participated in the study.	
				Mean participant age: 21.1 ± 1.4 years, 47.5% were female. Most participants (83.1%) self-identified as Caucasian, 16.9% as Black or African American.	
				Mean A1C level: 8.5±1.7%	
				Most participants received a diagnosis before 12 years of age (83.1%).	
Iversen *et al.*,^36^ 2019, Norway	Semi-structured interviews were conducted between November 2016 and January 2017.	How young adults with T1D experienced the transition from pediatric to adult health care services	An outpatient clinic at a large hospital in western Norway	11 (6 males, 5 females) of the 63 eligible young adults agreed to participate and signed the consent form before the interview started. The participants were aged between 19 and 23 years, had been living with diabetes for 9–19 years, and transitioned to adult care between 1 and 4 years before the study took place.	The study showed that the existing routines for transfer between pediatric and adult care are not optimal. The participants expressed that they were not prepared for the dissimilarities in follow-up and were predominantly less pleased with the adult care follow-up.
	Data were analyzed using interpretive description, an inductive approach inspired by grounded theory, ethnography, and phenomenology, and specifically designed to explore phenomena in clinical practice aiming to generate new knowledge and skills.				
Ladd *et al.*,^43^ 2022, Canada	Semi-structured interviews with adolescents (aged 17 years) with T1D at an academic institution from April 2017 to May 2018	Experiences and perceptions of a large and diverse group of adolescents with T1D during the peri-transfer process to understand the factors needing improvement during the transition period	Pediatric diabetes clinic at the Montreal Children’s Hospital of the McGill University Health Centre from April 2017 to May 2018. At the time of this study, transfer to adult care from Montreal Children’s Hospital was mandated at age 18 years. The transition process was not formalized, with pediatric endocrinologists individualizing plans for each adolescent during their teenage years. Clinics to which individuals were transferred included both general adult endocrinology clinics and diabetes clinics specifically for young adults, although the latter was less common. Quebec has universal health care for children and adults, which includes full coverage of basic diabetes supplies and insulin pump therapy (if started before the age of 18 years).	61 participants, 36% were male	Findings suggest that novel transition programs addressing changing interpersonal relationships, disease-specific self-management (adapted for age of diagnosis), and health care system navigation, supported by parents and peers, may be needed to improve transition care for adolescents with T1D.
	Coding followed the principles of thematic analysis			Mean age of T1D diagnosis: 9.0 years (SD 3.8 years)	
				Median average HbA1c: 70 mmol/mol (8.6%)	
				IQR 65–79 mmol/mol (8.1%–9.4%)	
				Participants had their final visit at the pediatric diabetes clinic at a mean age of 17.9 years (SD 0.2 years), and 93% had their first adult clinic visit within the following 6 months. The majority (72%) were seen in general adult endocrinology clinics, with only 11 participants seen in specialized young adult clinics. The clinic at which 6 participants were seen after transfer was not documented.	
				Participants were recruited through convenience sampling.	
Laursen *et al.*,^38^ 2024, Denmark	Data were collected by semi-structured interviews analyzed using an interpretative phenomenological analysis approach	To explore how young people living with T1D experience interactions with their care providers, and what it means for their developmental transition	The study was conducted in a youth clinic in Denmark. The youth clinic ensures that diabetes care is provided at the same location and with joint management across pediatric, youth, and adult care. A central feature of the youth clinic is that the young people keep their diabetes nurse from pediatrics, but get a new endocrinologist specialized in adult care. The youth clinic provides checkups every third month. In 2021, almost 250 young people visited the clinic.	10 participants (7 women and 3 men) aged 18–20 years	Continuity in the relationship with the diabetes nurse makes the transition from pediatric to adult care more satisfying and seamless. To support the developmental transition, care providers should gradually involve young people more in diabetes management and be supportive as they become more independent during the developmental transition.
				Diabetes duration: 5-13 years	
Leung *et al.*,^50^ 2021, Canada	Five focus groups on adolescents T1D nearing the age of transition to adult care	Adolescent perspectives, expectations, and preferences on program design in the transition to adult care	An urban academic pediatric hospital in Canada, within a universal health care system that does not currently have an adolescent transition program	Out of 96 eligible adolescents with T1D, 22 participated (23% participation rate), mean age 17.2 ± 0.9 years, diabetes duration 9.3 ± 4.3 years	The design of future transition interventions for adolescents with T1D should address the issues of individualization, identity, interconnection and impediment. Collaborative processes between pediatric and adult providers were also rated as important strategies to facilitate the transition to adult care.
	Thematic synthesis used for analysis				
Lundin et al.,^40^ 2007, Sweden	Participant observations of patient visits to nurses and physicians and 10 semi-structured interviews with care providers in 2 pediatric and 2 adult clinics in Sweden	To explore how care providers handle the transition process from pediatric to adult diabetes outpatient clinic and to describe their perception of adolescents’ needs during this process	Conducted (2005) in 4 different outpatient clinics in Sweden, 2 in pediatric and 2 in adult diabetes care	51 focused participant observations were conducted by attending patient visits to diabetes care providers	The way participating clinics handle transition greatly influences the process. Professional meetings appeared to be of vital importance to build bridges between pediatric and adult diabetes care in this study.
	Data were analyzed using the constant comparative method developed in the grounded theory tradition		2 clinics located in a university hospital in an urban city and 2 in a regional hospital in a smaller city	Total observation time was 100 hours: approximately 60 hours of visits and group sessions, with the remaining time in the waiting room area or together with care providers in relation to planned visits	
McDowell et al.,^46^ 2020, USA	Data were collected by online surveys (December 2017 to February 2018)	Barriers and facilitators adolescents face during their emerging adult years with T1D	A convenience sample of individuals with T1D were recruited from a private diabetes-specific Facebook group and Twitter to participate in an online survey	25 participants (males = 4, females = 21) were eligible and included in the study Participants were primarily Caucasian (n = 23, 92%), with mean age of 28 ± 3.2 years, and highly educated with either an undergraduate or graduate degree (n = 21, 84%). The participants’ mean age at diagnosis with T1D was 9 ± 4.7 years.	Individuals with T1D face a variety of challenges as they transition from pediatric to adult care providers. A proactive approach in educating adolescents is needed.
	A thematic analysis, guided by the Framework for Emerging Adults with T1D was used to explore the free-text data				
Nakhla *et al.*,^59^ 2017, Canada	Semi-structured interviews by phone (English or French) between June 2015 and November 2015	Pediatric diabetes care providers’ perceptions of transition care (perceived barriers to successful transition care, optimal methods to transitioning care, and measures of successful transition)	Only 3 (25%) of the participating centers had a formal uniform transition policy	Pediatric diabetes care providers from 12 diabetes centers in Quebec	Important gaps in transition care practices persist. Efforts should focus on improving education in transition practices for pediatric care providers and establishing formal transition policies and structures at the institutional level.
	Thematic synthesis used for analysis		There was a near universal absence of formal transition care readiness and planning, whether university-based or community-based. Only 3 centers (25%) used structured transition readiness and self-care skills assessment tools and regularly developed, updated, and documented patients’ progress and goals.		
Olsson *et al.*,^37^ 2023, Sweden	Semi-structured interviews were transcribed and analyzed using qualitative content analysis	To explore young adults’ experiences of living with T1D in the transition to adulthood, including experiences of the transfer from pediatric to adult care	Participants, having transferred from pediatric to adult care, were recruited at their regular diabetes clinic in Sweden from spring 2021 to spring 2022.	10 participants: 6 women, 4 men, aged 19–29 (median 22) years	In health care, it is important to emphasize not only diabetes-related factors but also emotional and psychosocial aspects of life connected to the transition to adulthood, including the transfer to adult care. The young adults wished to be seen as unique persons in health care during their emerging adulthood and should therefore be supported to achieve self-reliance through personal preparations for new challenges and for the consequences of transitioning to adulthood. Specialist nurses can provide appropriate knowledge and leadership.
				Recruited using purposeful sampling at diabetes clinics in university hospitals in Sweden	
				Participants were diagnosed at age 8–16 (median 11) years and had T1D duration of 5–19 (median 11) years.	
				They lived in both rural areas and cities. Eight participants had moved from their parents’ home and 2 had not; 5 participants were working and 5 were university students.	
Perry *et al.*,^13^ 2012, Australia	Semi-structured telephone interviews were conducted in 2008	The experiences of young adults with T1DM accessing adult diabetes services	4 rural locations in New South Wales, Australia:	26 people with diabetes (14 females, 12 males), with the mean age of 24.2 ± 2.4 years and mean diabetes duration of 13.3 ± 5.3 years	This was a first view of rural young people’s experiences with adult diabetes services. Reported experiences were in line with previous reports from other settings in that they did not perceive services in this rural area of Australia as meeting their needs; suggestions for service redesign differed.
	Data were analyzed by framework analysis		1: Weekly general adult diabetes clinic with a specialist physician, 3 DNEs/nurse consultants, a dietitian, and an ophthalmologist. Individual appointments with DNEs and dietitians available. Resident physicians in town.	Participants were significantly less satisfied with adult than pediatric services, with mean (SD) ratings of 63.9 (27.8) compared with 85.2 (13.9) (F = 545.523, *P* < 0.001)	
			2 and 3: Joint services provided by 2 DNEs and a dietitian, based on individual appointments. No specialist input, no resident or visiting endocrinologists. Resident general physicians in town, but none with a special interest in diabetes	Participants were recruited through a convenience sample.	
			4: Diabetes clinics cater for all ages, including children, serviced by a nurse and 2 community dietitians, but often lack experienced staff. Patients are generally managed by a primary care physician/general practitioner. The local Division of General Practice runs a voluntary database and recall system. No resident or visiting adult physicians.		
Pritlove et al.,^41^ 2020, Canada	Semi-structured interviews were performed and thematic coding was derived using a constant comparative approach	The experiences, perspectives, and needs of parents of emerging adults living with T1D during the transition to adulthood and adult health care services	Participants were recruited from young adult diabetes clinics in 2 tertiary care hospitals in Toronto, Canada, and through Diabetes Canada (a national diabetes charity)	16 parents (13 females and 3 males) participated in the study; mean age and range was 53 years	Adult HCPs should recognize both the ongoing involvement of parents in the ‘self’-management of emerging adults with T1D and the unique aspects of the caregiver burden that they experience.
				Their children ranged in age from 18–23 years and had been living with T1D for an average of 12 years (range 4–20 years).	
				Participants were recruited using a purposive sampling approach.	
Pyatak *et al.*,^54^ 2014, USA	Semi-structured interviews conducted in participants’ homes when possible (n=15), or the clinic or researcher’s office when necessary for logistical reasons (n=5)	Challenges contributing to disruptions in care during the transition from pediatric to adult care among young adults with T1D who are primarily in ethnic minority groups and have low socioeconomic status	Diabetes clinic in a public health care system in California	Out of 24 eligible patients, 20 participated in the study, with a mean (SD) age of 21.1 (1.1) years, mean (SD) HbA1c level of 95 mmol/mol (10.8 [2.4] %), and had been without routine diabetes care for a mean (SD) of 12 (10.4) months.	A confluence of challenges, including stressful life circumstances, health care system barriers, and the developmental trajectory of young adulthood, contributes to a high risk of loss to follow-up and poor health in this population of young adults with T1D. An integrated approach to transition addressing medical and psychosocial needs may facilitate improved follow-up and health outcomes in clinical settings.
	Analysis was conducted using a narrative thematic approach		No participants had private health insurance and most were of low socioeconomic status. Most participants had previously been insured through entitlement programs such as California Children’s Services, a state program for children with chronic health conditions, and MediCal, California’s Medicaid program. These programs are automatically discontinued at age 18 years, although young adults can reapply for continuing coverage through to 21 years.		
Ritholz *et al.*,^45^ 2014, USA	5 focus groups stratified by current level of glycemic control (A1C) were conducted	Perceptions that emerging adults with T1D have of their patient-provider relationships across the transition from pediatric to adult care	A comprehensive diabetes center in the northeastern United States	5 x 75-minute focus groups	Findings highlight the importance of enhanced provider awareness of T1D emerging adults’ complex feelings about the transition in care. Improved integration of individual- and family-centered approaches to develop mentally tailored diabetes care is needed to augment patient and provider relationships.
	Data analyzed using thematic analysis		Although the diabetes center has both pediatric and adult units, there is no established structured transition of care program.	Each group consisted of 3–7 patients (n = 26, mean age 26.2 ± 2.5 years, diabetes duration was 16.3 ± 4.7 years, mean age at transition from pediatric to adult care 20.3 ± 3.2 years, 62% female, 85% with college degree, 81% non-Hispanic White).	
				Groups were formed based on participants’ current level of glycemic control (A1C): lower A1C ≤ 8.5% and higher A1C >8.5%.	
				3 groups had lower A1C values (mean for lower A1C groups = 7.4% ± 0.6%), and 2 groups had higher A1C values (mean = 9.8% ± 1.0%).	
				Purposive sampling strategies used to identify English-speaking emerging adults with T1D who were diagnosed ≤18 years old and had T1D for at least 5 years.	
Simms et al.,^56^ 2017, USA	Data were collected by semi-structured interviews and were analyzed using conventional content analysis for broad themes	Experience of emerging adults regarding communication with pediatric diabetes HCPs, engagement in diabetes medical visits, and readiness for transition to adult diabetes care	Interviews were conducted in person after a diabetes medical visit or over the phone, based on participant preference.	Interviews were conducted with 20 emerging adults (mean age = 18.8 ± 1.5 years), who had been diagnosed with T1D for an average of 9.6 years (±3.6 years). The mean HbA1c of the sample was 8.5%, which is above the recommended A1C level for youth (A1C < 7.5%; American Diabetes Association, 2017). At the time of the interview, 19 out of 20 participants (95%) were receiving T1D care at a pediatric center, and 1 had recently transferred to adult medical care when the interview was conducted.	Findings support the conceptual model of communication and inform clinical implications for working with emerging adults with T1D. Continuity of care should be prioritized with transition-age patients. Additionally, HCPs should initiate conversations about engagement in risky behaviors and transition to adult medical care, and ensure emerging adults have time without parents to discuss these sensitive topics. Psychologists can enhance the transition process by facilitating effective patient–HCP communication and coaching both patients and HCPs to ask questions about risky behaviors and transition to adult medical care.
		Eemerging adults’ perspectives on health communication in pediatric care and the transition to adult care			
Tremblay *et al.*,^49^ 2020, USA	Semi-structured interviews conducted in a tertiary care U.S. pediatric center	Expectations of transition to adult care and experiences with transition planning among adolescents and young adults with T1D and an A1C >9%	A comprehensive pediatric diabetes center operating within a referral pediatrics hospital in Boston, MA, USA	14 subjects participated: 9 adolescents and 5 young adults	A lack of transition preparation and anxiety about transition and adult care among youth with T1D and elevated A1C.
	Thematic synthesis used for analysis			Mean age 17.1 ± 3.2 years	The results may help guide early, iterative pediatric transition counseling, with a special focus on addressing attachment and fears about adult diabetes care.
				57% male	
				79% Caucasian, 14% Hispanic	
				Diabetes duration 8.2 ± 4.6 years, mean A1C 10.0% ± 0.8% for adolescents and 10.1% ± 0.7% for young adults	
Vaillancourt *et al.*,^48^ 2023, Canada	Data collected through semi-structured interviews and analyzed using inductive thematic analysis	To explore the post-transfer perceptions of emerging adults living with T1D relating to their transition to adult care	Participants were recruited through the Montreal Children’s Hospital Paediatric Diabetes Clinic in Canada over a 12 month period (April 2017 to May 2018).	33 emerging adults living with T1D recruited by convenience sampling and contacted for a semi-structured interview post-transfer to adult care (16.2 ± 4.2 months post-transfer)	The experiences and perceptions of emerging adults are invaluable to guide the ongoing development and improvement of transition programs for childhood-onset chronic illnesses.
			Semi-structured interviews were conducted after their transfer to adult care between March 2019 and October 2019.	Mean age of 19.28 (±0.42) years	
			Participants were transferred to: adult care clinic with a different care team (19; 57.6%); adult care clinic with their pediatric doctor (5; 15.2%); young adults’ transition clinic (9; 27.3%).	13 (39.4%) of sample were males	
				Mean HbA1c: 9.37 (±2.16)	
Visentin et al.,^51^ 2006, Australia	Interviews conducted between February 2004 and June 2004	Concerns and issues of key diabetes health delivery stakeholders about current service delivery and ways to improve same, and the perspective of adolescents living with T1D and their experiences surrounding the process of transition	Adolescents recruited from 2 major children’s services located in metropolitan Adelaide	Interviews with 21 diabetes health professionals and 10 adolescents, aged 15–18 years, who had been diagnosed with T1D for a minimum of 12 months.	The project findings have set the scene to establish a multidisciplinary working party to work collaboratively across agencies to develop effective transition pathways.
	Interview data were generated and analyzed using a response focus framework provided by fourth-generation evaluation research		Of the health professionals, 7 were medical consultants: endocrinologists, pediatricians, pediatric endocrinologists, and physicians (n=7), 10 were DNEs (n=10), 3 were dietitians (n=3), and 1 psychologist (n=1). All interviews with health professionals were conducted at the health service site in which they worked. Eight interviews with adolescents were conducted at the diabetes clinic and 2 were undertaken in the adolescents’ own home.		
Walsh *et al.*,^55^ 2018, Ireland	Data collected by semi-structured interviews and analyzed by thematic analysis	The perceptions of young adults, parents of young adults, and HCPs of the transition process for young adults with T1D in the West of Ireland	In each of the 3 participating hospitals, the transition process typically occurs the summer after the leaving certificate over 3 clinic visits; firstly, the adult staff attend the pediatric clinic, then the pediatric staff attend the adult clinic, and finally the adolescent joins the adult diabetes clinic. However, there are some variations between the hospitals. In the first hospital, where there are 6–12 transitions per year, the adolescents transition from a teen clinic to a young adult clinic that have a common ANP. In the second hospital, transition occurs in an ANP-led young adult clinic, with 5–10 transitions per year. In the third hospital, transition involves only a change in consultant with 5–6 transitions per year.	A total of 20 participants were recruited, representing a good mix of the different groups across the 3 sites (n=20); 6 young adults (2 from each of the different hospitals); 7 parents of other young adults; and 7 HCPs. The HCPs included 1 ANP and 2 doctors from the first hospital, 1 DNS and 1 doctor from the second, and 1 DNS and 1 doctor from the third.	This study highlights the importance of encouraging adolescents’ autonomy in the years leading to transition. A key HCP link between both services appears to facilitate smooth transition. Being flexible and supportive of both parents and adolescents, including the provision of mental health services, are other important considerations.
				Purposive sampling was used to recruit participants from the 3 stakeholder groups in each of the 3 hospitals.	
Williams *et al.*,^53^ 2021, Canada	Mixed method study	How transition is occurring across the jurisdiction and identify methods for improving clinical services for pediatric patients with a chronic condition during their move into adult care	4 regional health authorities in a Canadian province (Newfoundland and Labrador [NL]) with a small, mostly rural population and high rates of T1D	15 interviews conducted with pediatric and adult diabetes providers, representing providers from all 4 health regions	There are different approaches for transitioning patients with diabetes into adult care across NL. This variation may not negatively impact patient outcomes, particularly in rural areas. The approach of combining reviews of administration data with a detailed analysis of current processes could be employed in other jurisdictions to identify appropriate quality improvement initiatives.
	Qualitative data collected via semi-structured interviews (October 2017–July 20)		Various models of transition care are being employed, reflecting staff and resource availability in different centers. While no formal transition program was identified in either region, some providers, particularly in rural areas, reported being comfortable with their current transition practices.		
Thematic analysis used for qualitative data analysis			

ANP, advanced nurse practitioner; DNE, diabetes nurse educator; DNS, diabetes nurse specialist; HbA1c, hemoglobin A1C; HCP, health care provider; TD1, type 1 diabetes

## Appendix IV: Study findings and illustrations

**Table:**

Allen *et al.*,^52^ 2011	
Finding	The transition from child to adult services (C)
Illustration	‘I’m involved when something is out of the ordinary, if it’s the ordinary she just gets on with it, if she thinks something is a bit different to what she’s expecting then we’ll discuss things with her.’ (4-M50, a mother)^(p.997)^
Finding	The ambiguous positioning of mothers (U)
Illustration	‘I don’t know what’s expected of me anymore and I don’t know what to expect from them…he’s an adult and they keep saying that…but for me he’s still my son and he’s still got diabetes and he still needs help and guidance.’ (1-M137, a mother)^(p.997)^
Butalia *et al.*,^47^ 2020	
Finding	Communication technology (U)
Illustration	“I think a text message or an email would sort of remind you to keep on track.” (Group 1, an adolescent)^(p.4)^
Finding	Transition and diabetes education and preparation (U)
Illustration	“…and I know at the Children’s they were like you need to learn to do carb counting but my Mom did everything for me. She still helps me, but I think now, having an educational class to make sure that us as 17-year-olds actually do know how to manage our diabetes on our own.” (Group 2, an adolescent)^(p.4)^
Finding	Social and peer support (U)
Illustration	“I feel like ya know and I think that ya know, it is helpful to be able to talk to someone that at least understands what you are going through.” (Group 3, an adolescent) ^(p.5)^
Garvey *et al.*,^42^ 2014	
Finding	Nonpurposeful transition (U)
Illustration	There really wasn’t much discussion about it. Other than where I was going to go. Yeah, I just kind of had to take it from there. [female, age 25]^(p.194)^
Finding	Vulnerability in the college years (U)
Illustration	All throughout college, the biggest thing I struggled with is balancing my health care and social life. [female, age 23]^(p.194)^
Finding	Unexpected differences between pediatric and adult health care systems (U)
Illustration	The most shocking thing was coming in the first time, and it wasn’t the standard procedure I was used to. Yeah, it’s just something you have to kind of pick up […]. It was just very different. [female, age 25]^(p.194)^
Finding	Patient wish list for improving the transition process (U)
Illustration	If I could make a recommendation to [pediatric providers] regarding pediatric care it would be, even if they only have one adult provider that they recommend, have somebody that you recommend […] I wish that I had received that from my pediatric care […] just something as simple as that would have made a world of difference. [male, age 27]^(p.195)^
Guitard-Munnich and Houdan,^57^ 2019	
Finding	Fatality, passivity, and waiting (U)
Illustration	“It’s inevitable” (A young adult)^(p.414)^
Finding	Repetition (NS)
Illustration	None
Finding	Family and hospital (C)^(p.415)^
Illustration	“Helping the little ones make them feel less alone” (A young adult)^(p.415)^
Finding	Parental anxiety (U)
Illustration	“They get a real kick out of it, they dramatize because they don’t know” (A young adult)^(p.416)^
Finding	Availability (NS)
Illustration	None
Finding	Care organization (C)
Illustration	“You have to go with the flow” (A young adult)^(p.416)^
Finding	Relationship with other young people (U)
Illustration	“I don’t want diabetes between me and my friends” (A young adult)^(p.416)^
Finding	What teenagers project when they go to the care for adults (U)
Illustration	“There won’t be that little extra” (A young adult)^(p.416)^
Finding	The failures (NS)
Illustration	None
Finding	The patients perspectives and suggestions for improving the transition and avoiding break-ups (NS)
Illustration	None
Hansen and Jensen,^58^ 2017	
Finding	Transition unawareness (U)
Illustration	I don’t think it was something we talked about. I think it was perhaps the last consultation or the one before that, they mentioned, that it was time for me to move. There wasn’t been any communication about it. I don’t think, there was. (U10, an adolescent)^(p.54)^
Finding	A crucial change in relationship with health care providers (U)
Illustration	What’s relevant for me and for you is my diabetes and my insulin pump. If I have any other questions and questions on my blood sugar. That’s what relevant. In the pediatric ward, it was more like; what are you doing in your vacation and so on. There is less of that here, and that’s fine by me. (U1, an adolescent)^(p.56)^
Finding	Partnership without shared decision-making (U)
Illustration	Usually you agree with the doctors. They know what is best, besides yourself, of course. Usually you agree. I have never tried to say no – I don’t think that’s a good idea. You make sure, you agree and usually that’s what you do. (U1, an adolescent)^(p.57)^
Harris *et al.*,^39^ 2020	
Finding	Working with what is available (C)
Illustration	… the majority of their [General Practitioner] caseload is older people with diabetes …. where it’s so much about complications management. Their lifestyle advice that they are used to giving is not lifestyle advice that suits a teen or young adult lifestyle. (HCP:1)^(p.338)^
Finding	Mapping a route to better services (U)
Illustration	I think if you really want to target young people with type 1 diabetes, you do not say that an 18-year-old is the same as a 30-year-old. I think they need separate group sessions where you can target the different age ranges or different developmental stages. (HCP:6)^(p.339)^
Hilliard *et al.*,^44^ 2014	
Finding	Timing of transfer (U)
Illustration	“He can stay here [in pediatric care] forever. For my money, he doesn’t have to go anywhere. I’d like to see him be a bit older.” (mother to 16-year-old male)^(p.350)^
Finding	Early transition preparation (U)
Illustration	“Well, she’s mentioned how when she goes away to college, how is she going to keep track of her numbers at night, and how is she going to manage that. So, she’s already anticipating that, which I think is great.” (mother of a 16-year-old female)^(p.350)^
	“[Preparation for transition could have been improved if I]…could have slowly been doing things on my own, that way I would already be used to going to the doctors by myself and telling him what was going on.” (19-year-old female)^(p.350)^
Finding	Developmentally appropriate interaction (U)
Illustration	“I was finally being treated like the adult I should have been. At the age of 17 I feel I was too independent to have [my diabetes center] ‘holding’ my hand through diabetes care. I think once a human matures they no longer need their doctors to treat them like they are 10 years old.” (20-year-old male)^(p.350)^
Finding	Social/family support and building a “safety net” (U)
Illustration	“I lost the constant support and attention from my parents that I had always had. I still had support from my parents, friends, and doctor, but overall I had to learn to handle my diabetes independently and take more responsibility for myself and my health, and that was very difficult.” (21-year-old female)^(p.350)^
	“I think the challenge at college is just going to be not having the safety net and the transition with mom and dad, and not having the friends that she has known and her friends who know her diabetes….Mom and dad aren’t going to be there every day saying, ‘What’s your blood sugar? What’s your bolus?’ And I just think it’s going to be a huge, huge, huge shift in responsibility when she realizes.” (mother of 16-year-old female)^(p.350)^
Finding	Coordinating care (U)
Illustration	“Having appointments where both the pediatric and adult care professionals would be there to meet and discuss my treatment.” (21-year-old male)^(p.351)^
Iversen *et al.*,^36^ 2019	
Finding	Limited information about the transition (U)
Illustration	I cannot quite remember, but I do not think I received any information. I received a letter about the next appointment at the adult outpatient clinic. (a young adult)^(p.726)^
Finding	Transition from a frequent, thorough and personal follow-up to a less comprehensive and less personal follow-up (U)
Illustration	Yes, because I feel like the doctor is just measuring you, and then you leave again. However, the disease is about so much more than taking insulin. The disease affects you mentally. Therefore, I think the focus on how you are really doing is less evident in the adult outpatient clinic. At the paediatric outpatient clinic, they asked me how I felt and things like that. (a young adult)^(p.726)^
Finding	The importance of being seen as a whole person (U)
Illustration	Now, I feel in a way that talking about diabetes is a personal thing and how I have handled it or are handling it is personal. It would be easier to talk about it with people I have met before than with total strangers. (a young adult)^(p.727)^
Finding	Limited expectations of how the health care services were organized (U)
Illustration	I did not feel I had any other choice really. Because at the GP you can get check-ups more often. I was told that there would not be as many follow-ups after the transition to the adult outpatient clinic, so I should get a GP where I could get my mean blood glucose checked more often. (a young adult)^(p.727)^
Ladd *et al.*,^43^ 2022	
Finding	Shifting of diabetes management from parent to child (U)
Illustration	Not back off cause that’s [kind of] harsh but like a little bit back off … I feel like even if I make mistakes like I’ll learn from them more than if my parents are always [kind of] bothering me about it … [I kind of] need to do that by myself. (Participant 18, an adolescent)^(p.5)^
Finding	Attachment to paediatric healthcare team (U)
Illustration	I’m actually kind of sad that I’m leaving the team, they were so great. (Participant 12, an adolescent)^(p.6)^
Finding	Worry about future relationship with adult healthcare team (U)
Illustration	My concern mostly would be that [in adult care] we would lose a little bit of the personal connection with the doctors. [In paediatrics] I feel like it’s a lot more, it’s warm, and it’s very friendly, so … I’m scared of it being very cold. (Participant 75, an adolescent)^(p.6)^
Finding	Adaption to new diabetes care responsibilities (U)
Illustration	I will be overwhelmed, you can say that, cause sometimes when I’m going out, I usually call my mom to say, because she has the chart at home, so I will always tell her what, how much should I do, but the fact that in 3 months I will be 18 so everything’s on me …. It’s gonna be overwhelming at first but not that much if I get the hang of it. (Participant 48, an adolescent)^(p.6)^
Finding	Age of diagnosis affecting comfort with self-management (U)
Illustration	Of course, it’s different maybe for other kids if you’ve been diagnosed when you were like 14 … and you’re not used to living with it whereas if you’re diagnosed at 5 like I was and it’s part of you, so for me there’s definitely like less that I have to learn. (Participant 13, an adolescent)^(p.6)^
Finding	Perceived responsibility of paediatric healthcare team (C)
Illustration	Obviously I don’t know anything about like, if there’s any differences, what they have here [in paediatrics] and there [in adult care] so it would be good to know, understand what it’s like. (Participant 36, an adolescent)^(p.7)^
Finding	Perceived self-responsibility (U)
Illustration	I have to be more responsible, more consistent, doing more blood sugars, more testing, so, basically managing […] my diabetes a bit better because I’m gonna be alone (Participant 53, an adolescent)^(p.7)^
Laursen *et al.*,^38^ 2024	
Finding	Continuity in the relationship with the diabetes nurse (U)
Illustration	She [the diabetes nurse] has been with me from the beginning, so she knows that I can manage diabetes… but she also knows that I sometimes fail to manage it. But she knows what can get me on the right track (Sophie, female, a young adult).^(p.130)^
Finding	A personal patient–provider relationship (U)
Illustration	My doctor sometimes says I should try scuba diving, he has tried it a lot, and then we talk about many different places. I went to Cuba and then he said, ‘I really want to go there’. We talk about personal things (…) I think it is very nice and it makes the check-ups less serious and creates a homelier atmosphere (Anna, female, a young adult).^(p.130)^
Finding	Increased level of personal responsibility and diabetes management skills (U)
Illustration	When my blood sugar is very unstable, we analyze ‘what did I do that day’, and ‘what do you think about the fluctuation right there’ (…) ‘okay, everyday about 7.30 p.m. you are high [in blood sugar level], it can be explained by dinner. Then maybe you should take some more insulin earlier’… We are looking for patterns. (a young adult)^(p.131)^
Finding	Practical and emotional support (U)
Illustration	We read off my insulin pump and so on. I was not at the hospital, but we just talked on the phone (…) It worked out very well that you were always in contact with someone, so you did not have to deal with it [measure blood sugar levels] by yourself. (a young adult)^(p.131)^
Leung *et al.*,^50^ 2021	
Finding	Having choices in the transition experience (U)
Illustration	“Different people learn differently, like videos would help some people, having the information in front of you would help others. Being able to email that doctor—like having different options—so it’s not just everybody’s going down this one path.” (Participant 1, Focus Group 1, an adolescent)^(p.5)^
Finding	Meeting adult provider before transition (U)
Illustration	“Maybe like for [age] 17–19 you’re meeting with your children’s doctor and the doctor you’re going to after, so they can talk about what’s best for you and so they can learn about you together so that your children’s doctor can talk to your new doctor about how he’s found or she’s found best ways to help you.” (Participant 1, Focus Group 1, an adolescent)^(p.5)^
Finding	Specific transition preparation (U)
Illustration	“A longer heads up maybe because I feel everyone was just telling you in the last visit to, ‘Hey, you pick a doctor.’ Maybe 5 visits earlier. Making sure you know it’s coming.” (Participant 9, Focus Group 2, an adolescent)^(p.5)^
Finding	Stigma of type 1 diabetes (U)
Illustration	“I’ve gotten kicked out of restaurants before … I was very embarrassed at the time. I’m not doing anything wrong, but I can’t really explain because I’m being pushed out of a restaurant. And it wasn’t really worth it at that point to be like, ‘No, like I’m a type 1 diabetic.’” (Participant 14, Focus Group 3, an adolescent)^(p.5)^
Finding	Disclosure of diabetes diagnosis (U)
Illustration	“There’s another diabetic at my school… on a pump… He was just typing, this was during one of my… mock exams and the teachers were like, ‘…Why are you on your phone? I thought you handed in your phone?’ And he was like, ‘I’m diabetic…’ Obviously you could tell that he wasn’t comfortable with the whole class knowing.” (Participant 19, Focus Group 5, an adolescent)^(p.5)^
Finding	Confusion with type 2 diabetes (U)
Illustration	“When they bring up diabetes, they don’t know there is a difference between type 1 and type 2. It’s more just there’s diabetes in general. It can be a little bit frustrating sometimes that stigma around type 2 diabetes.” (Participant 12, Focus Group 3, an adolescent)^(p.5)^
Finding	Resilience and self-advocacy (U)
Illustration	“It gets annoying cuz I’ve gotten it so much, but I try not to umm be like annoyed with people for being curious. There’s nothing wrong with being curious. But then also like I’ve gotten my explanation talk down to 30 seconds, you know, for what diabetes is.” (Participant 18, Focus Group 5, an adolescent)^(p.5)^
Finding	Peer support (U)
Illustration	“It’s never nice going through something all by yourself. Sure, your parents are there, and they love you, but they don’t know what’s going on. They don’t know how to make you feel better. But somebody who’s going through the same thing knows exactly how you’re feeling.” (Participant 1, Focus Group 1, an adolescent)^(p.5)^
Finding	Near peer support (U)
Illustration	“I’d say it might have been helpful to have some sort of I dunno phone number for questions or something or maybe even just I dunno some sort of contact with somebody who has done this before and kind-of knows how it went.” (Participant 21, Focus Group 5, an adolescent)^(p.5)^
Finding	Parents empowering autonomy (U)
Illustration	“Yeah, my parents have slowly started stepping back. So, like now I’m monitoring when I’m low on reservoirs or site changes or anything. So, it’s kinda nice, but it’s also like kinda hard to keep track of. It’s slowly piling up. Like everything you have to keep track of. But they’re taking it slow. And they’re slowly letting me take over responsibilities, which is kinda nice.” (Participant 11, Focus Group 3, an adolescent)^(p.5)^
Finding	Loss of bond with paediatric team (U)
Illustration	“Umm well personally, I’ve known my doctor since I was like 5. Umm I feel like I have a bond with them and I feel like going to a new doctor I won’t be as comfortable talking about some of the things that could be important that I might not necessarily think as that important but could turn out to be serious later.” (Participant 5, Focus Group 2, an adolescent)^(p.5)^
Finding	Fear of not having bond with adult team (U)
Illustration	“For me, definitely for the doctor, I want somebody who is social and isn’t there to just be a doctor. I want somebody there who wants to learn about me, to kinda get to know me, kinda be like a friend, so it’s not such a blatant appointment about this needs to be better, this needs to change, this – so it’s not just all about diabetes.” (Participant 1, Focus Group 1, an adolescent)^(p.6)^
Finding	Self-care takes work and time (U)
Illustration	“Whatever you are doing, you have an extra hurdle… Whenever I was trying out for soccer teams, it would be such a hassle if I had to deal with my sugars or something. It would be such a setback. It’s a little hard to deal with. It’s annoying… It’s something else that you have to stress about. It’s something else that you have to deal with. Whatever someone else has to do, you have to do more.” (Participant 15, Focus Group 4, an adolescent)^(p.6)^
Finding	Unpredictability and restrictiveness (U)
Illustration	“You always have to be extra prepared… For example, you’re on the road, there’s no stops, you can’t just like stop and get a pop if you go low or like on an airplane you can’t really, you can, but like…” (Participant 22, Focus Group 5, an adolescent)^(p.6)^
Finding	Emotional burden (U)
Illustration	“I think people need to be more aware of mental health issues with diabetics as well cuz I found that every single friend… I do know who has diabetes… a lot of them have like depression, a lot of them have anxiety, a lot of them have certain things that they deal with… I feel that the clinic should talk more about that.” (Participant 17, Focus Group 4, an adolescent)^(p.6)^
Lundin et al.,^40^ 2007	
Finding	Uncertainty of appropriate strategies (U)
Illustration	‘‘they don’t listen to this just now …… they don’t think it is particularly important or interesting … I would like to find other ways to meet … perhaps it’s not the right time …’’ (I-A, a health care professional)^(p.4-5)^
Finding	Adjustment of strategies (U)
Illustration	“it would probably be good for the patients, to prepare and for us to know what they have been used to before, because we actually don’t know that, well I don’t anyway’’ (I-A, a health care professional).^(p.5)^
Finding	Bridging activities (U)
Illustration	‘‘It is important for us to hear how it has … what affects the adolescent and what their weaknesses … you can’t just say that they have neglected themselves but there are a number of problems which they have no control over … which makes it difficult and you should have an understanding of this as well when they come’’ (I-A, a health care professional)^(p.5)^
Finding	Deciding time for transfer (U)
Illustration	‘‘I think that there are quite a lot who almost beg and pray to stay and they bring up the question themselves of when they have to leave …’’ (I-P, a health care professional)^(p.5)^
Finding	Ending relationship in paediatric care (U)
Illustration	‘‘The young man came with his father for his first visit to adult diabetes care. He told me himself that they had already talked about it when he was 18 that he would transfer, but that there could be a long waiting time. When he hadn’t been given an appointment for 1.5 years, he rang to paediatrics himself because he needed a new prescription’’ (O-A, observation filed notes)^(p.5)^
Finding	Introducing adult care (U)
Illustration	‘‘The nurse, working in both settings, gives the phone number at both places and says that he will be given an appointment in September and perhaps to day-care group if he is interested sometime next year’’ (O-P, observation filed notes)^(p.6)^
Finding	Exploring knowledge and motivation (U)
Illustration	‘‘It’s not really the knowledge that is lacking, but the motivation to self manage and understand … there are so many other things at this age which is important to them’’ (I-P, a health care professional)^(p.6)^
Finding	Increasing contact desired (U)
Illustration	‘‘I think it’s good, now we get a totally different offer from the PDC thanks to the new nurse from paediatrics, it gives us so much more, there are so many things that we don’t always think about on the adult side and vice versa, we have already understood that the nurse has implemented certain things that no one thought about before’’ (I-A, a health care professional)^(p.6)^
Finding	Improving strategies after feedback (U)
Illustration	‘‘It is nevertheless quite encouraging if you find out that something of what you did has stuck … and that they have benefited from the support they received then, maybe it will turn out better in the long-term anyway … so it’s very important I think’’ (I-P, a health care professional)^(p.6)^
Finding	Need for organisational changes (NS)
Illustration	None
McDowell *et al.*,^46^ 2020	
Finding	Importance of support from key players (parents, peers and providers) (U)
Illustration	Me doing everything early on and having supportive parents really helped me to be independent (31 years old, female)^(p.697)^
Finding	Challenges of navigating the healthcare system (U)
Illustration	Young adults do not know what they are doing when they leave paediatric care. Many essential aspects of diabetes care, such as navigating finding a new doctor, getting prescriptions and dealing with insurance are complete mysteries. (25 years old, male)^(p.697)^
Finding	Mental health needs of teens with T1D (U)
Illustration	Doctors on your case when you are trying your best makes it hard to keep trying. I started giving myself a ‘burnout day’ every so often where I did not pay attention to diabetes so that I could feel ‘normal’. (28 years old, female)^(p.697)^
Finding	Managing day-to-day life with T1D (U)
Illustration	Hormones, activities and diet change during this time. These all account for variation in glucose regulation. (27 years old, female)^(p.698)^
Finding	Early independence to ease transition (U)
Illustration	Review the stuff that you teach diabetics at the beginning. I learned what caused diabetes before 3rd grade. Now I’m 26 and struggling to remember some of the basics. Help me to manage diabetes in all situations, which my endocrinologists did. This has given me the ability to manage the unexpected moments throughout college and my young adult life. (25 years old, female)^(p.698)^
Nakhla *et al.*,^59^ 2017	
Finding	Reliance on within-center referrals (NS)
Illustration	None
Finding	Lack of availability of adult providers and multidisciplinary team in the adult care setting (NS)
Illustration	None
Finding	Quality of pediatric care superior to adult care (NS)
Illustration	None
Finding	Vulnerability during the transition period (NS)
Illustration	None
Finding	Lack of understanding and appreciation by adult care providers of the specific challenges emerging adults face during the transition period (NS)
Illustration	None
Finding	Pediatric providers’ lack of time in planning for transition and lack of an identified staff person responsible for transition care (NS)
Illustration	None
Finding	Measures of successful transition (NS)
Illustration	None
Finding	Overlap of care between pediatric and adult healthcare systems seen as ideal method of transition care delivery (NS)
Illustration	None
Finding	Moving out of region (NS)
Illustration	None
Olsson *et al.*,^37^ 2023	
Finding	Life-changing disease onset (U)
Illustration	In the beginning, I think I found it a little difficult to take it all in and I did not think about the fact that it would be there for ever and permanently. Later, I was quite sad. (Participant 7, a young adult)^(p.4625)^
Finding	Not always straight forward (U)
Illustration	I can’t remember getting any information about how this [i.e., the disease] can limit us beyond the clinical picture—how it affects one’s well-being and what kind of everyday life limitations there can be. (Participant 9, a young adult)^(p.4626)^
Finding	Hopeful and challenging future (U)
Illustration	I believe that the disadvantages for me have been most substantial when I’ve been thinking about what I want to do in the future. I wanted to become a police officer once, but I was held back due to living with diabetes, and that was quite a great loss. (Participant 9, a young adult)^(p.4626)^
Finding	Not like everyone else (U)
Illustration	I perceive myself as more ill when I’m supposed to pick up a cannula, adjust the pen, and do injections in my stomach. I experience myself as sicker. I also believe it to be harder to do this among others, somehow. (Participant 7, a young adult)^(p.4626)^
Finding	Ambivalence regarding openness (U)
Illustration	It took a while for my friends in upper secondary school to even know it [i.e., that I was living with T1D]. I didn’t want it to define me anymore, and this was a chance to be “normal” again. (Participant 8, a young adult)^(p.4627)^
Finding	Efforts to make peace with oneself (U)
Illustration	I’ve never come to that [i.e., acceptance] since I didn’t choose to live with it. But anyhow, I’ve arrived at a certain point where I can kind of handle it. That was after my nursing education and it was around 17 to 18 years later [i.e., after the disease onset]. I manage, but I believe I will probably never accept it. (Participant 6, a young adult)^(p.4627)^
Finding	Early responsibility for self-management (U)
Illustration	I believe it [the responsibility for diabetes self-management] has slowly gone down that path all along. That’s exactly it, there’s more and more responsibility. And here and now, all the responsibility is on me. (Participant 8, a young adult)^(p.4628)^
Finding	Security with the familiar (U)
Illustration	I got it [i.e., the disease] at an early age. I suppose that’s better than later. I was already managing my diabetes when I got to that age [i.e., the transition to adulthood] for that reason. It was not a big deal—it was already part of my daily life. (Participant 3, a young adult)^(p.4628)^
Finding	Separation from family members but still in need of support (U)
Illustration	I know some people with T1D and when I meet them, it’s having somebody with the same disease as yourself. Somehow, you become different [when living with the disease], but when you meet these people, you’re some kind of “normal”. I see people who I can speak to. (Participant 4, a young adult)^(p.4629)^
Finding	Abrupt transfer between clinics (U)
Illustration	I’m happy about the way they treated me and that, it [i.e., the transfer] went well, but it felt really abrupt. Overnight, there were completely new people and despite them being very kind there’s like something completely new. Especially when there is such a difference between how the paediatric and the adult clinics work. If the transfer could proceed more slowly, it would not be as abrupt. (Participant 7, a young adult)^(p.4629)^
Finding	Standardized treatment despite individual needs (C)
Illustration	There was a great focus on everything that was regarded as positive at the paediatric clinic. But at the adult clinic, it was more like “Your HbA1c outcomes are bad and you are doing this and that wrong”. It led to me frequently leaving the clinic angry. (Participant 6, a young adult)^(p.4630)^
Finding	Importance of trusting, caring relations (U)
Illustration	We, who have T1D, come here regularly and the visits are not that personal, so maybe you [i.e., HCPs] could spend a little more time with us. Who we are, what kind of life we are living, and what kind of support we need, and so on. I’d desired that, not simply being a name on a paper. (Participant 1, a young adult)^(p.4630)^
Perry *et al.*,^13^ 2012	
Finding	Leaving paediatric services (U)
Illustration	In children’s… once every 4 months you’d get all the specialists come up from (city) hospital, which was really good. And you’d go along and they’d do your HbA1c and they would talk to you about it and you know, you felt like they were… at the top of the field kind of thing. But once you turn eighteen… all that kind of stuff, it’s like it just stops. (male, 27 years)^(p.1958)^
Finding	Access to health professionals (U)
Illustration	It seems a lot more like a general doctor’s visit now days, which makes it, you know it’s harder to get in to see them. (female, 27 years)^(p.1958)^
Finding	Accessing up-to-date clinical information (U)
Illustration	I’d like to have more specialists so that in the country you can get more of the newer things that come out. … it would be good to be able to go to a specialist and talk about the options that are, like insulin pumps and all that kind of stuff, but it’s just really not an option here. (male, 27 years)^(p.1959)^
Finding	Age-appropriate services (U)
Illustration	I personally don’t find (adult diabetes clinics) that useful… it’s really horrible to sit there and watch all the complications they have and what they’re going through. For me, who’s only 26, like that’s quite a big jump in age difference. There was no one my age there. It was all elderly people and it was always late. …. there doesn’t seem to be anything catering for my age group. (female, 26 years)^(p.1959)^
Finding	Fragmentation and lack of coordination (U)
Illustration	I’m good with my diabetes but I don’t like sitting in a room for 4 hours waiting for a doctor, going in and seeing him for 5 minutes and then leaving. There’s not really any point turning up. (female, 23 years)^(p.1959)^
Finding	Education (U)
Illustration	There doesn’t sort of seem to be a lot of help… for the people who are my age, which is probably, in my eyes, which is where you need to be starting to really educate ‘cause it’s now that I’m really listening, (laughs) ‘cause as a teenager they tell you lots of things that if you’re in that state of mind where you don’t take it on board. Whereas now, I take a lot more on board so it’s (laughs) probably more helpful now. (female, 26 years)^(p.1960)^
Finding	Social and peer support (U)
Illustration	Just getting together with other people my own age that have diabetes and seeing how they deal with it and having a gathering where you can get information and still have a good time…. partners as well… so that they could also learn more about managing it. (female, 22 years)^(p.1960)^
Pritlove *et al.*,^41^ 2020	
Finding	Parental experiences of transition (U)
Illustration	I kind of had a period of ‘what now’? You know, like I mean it was never my disease to deal with [but] now what’s my role? And figuring out you know, where I fit. (P3, a parent)^(p.3)^
Finding	Renegotiating parent–child roles, responsibilities and relationships (U)
Illustration	You want to give them enough rope so that they can learn things for themselves, but not enough to hang themselves with. (P6, a parent).^(p.4)^
Finding	New and evolving fears (U)
Illustration	I think now the biggest [fear] is that, all through the years you have some sort of control…and now I can’t tell you what his last A1C was. That’s all information that he keeps to himself…That was a big shift, to kind of relinquish control when I had so much control. (P3, a parent)^(p.4)^
Pyatak *et al.*,^54^ 2014	
Finding	Health provider/health system challenges (U)
Illustration	Jeremy: I called for an appointment to see a doctor…They said that they charged every time you go to an appointment… Eventually, we had to go to the emergency room to get insulin. And they were like, “So why didn’t you go to a doctor [at County]?” And I told them that they wanted to charge me. And they said, no, that is not true, that it is free. (Male, a young adult)^(p.1618)^
Finding	Developmental challenges (U)
Illustration	Karen: I do want to [get my HbA1c] below seven because I have had it high this past year. Well, this past month, just because of nursing [school], I was just so caught up, and I was disregarding my diabetes… It’s like out of balance, I guess. It’s just because I was so busy with school. It’s just the pressure from school. (Male, a young adult)^(p.1619)^
Finding	Psychosocial challenges (U)
Illustration	Roberto: If you go [to the clinic] and you say, I have just a little bit of insulin – because there’s a lot of people waiting to be seen by a doctor – they’re like, then you can come back tomorrow… [They say] come back tomorrow, and it’s going to be faster. And I was like, but I have to go to work, I have to support my family. (Male, a young adult)^(p.1621)^
Ritholz *et al.*,^45^ 2014	
Finding	Loss and gain in provider relationships across the transition (U)
Illustration	I think the hardest thing in health care… is that you build the relationship with the person…. That is why I didn’t want to leave [pediatrics]. Like I felt like they got me and like they totally knew what I was about, and that is one of the hardest things… about adult care is rebuilding that relationship. (female, 25 years old, transitioned at age 23)^(p.43)^
Finding	Partners in care versus on one’s own (U)
Illustration	It’s [adult care] less of the dictating and more of the collaboration, which has been good…. So, it’s more of a conversation rather than a, “this is what you have to do” type of thing. So that’s been helpful. (male, 24 years old, transitioned at age 18)^(p.43)^
Finding	Improving provider approaches to transition (U)
Illustration	I feel it might have been helpful to either have some sort of recommendation in place in terms of who I might want to see based on my history and medical details. I guess even just communicating with someone on the adult team to kind of bridge the transition beforehand. It doesn’t even necessarily have to be a physical meeting of everyone, just kind of communicating more. (female, 27 years old, transitioned at age 18)^(p.44)^
Simms *et al.*,^56^ 2017	
Finding	HCP interaction style (U)
Illustration	“She definitely does listen to me; it’s refreshing to have a physician who cares that much.” (A young adult)^(p.420)^
Finding	HCP consistency (U)
Illustration	“The fact that she recognizes me when I walk into the clinic, or remembers something I said in my last appointment, is nice to know that she actually knows who I am as a human being, and I think that’s really important.” (A young adult)^(p.420)^
Finding	Support for autonomy (U)
Illustration	“She gives me more options about how to treat things. I have more input.” (A young adult)^(p.420)^
Finding	Parental involvement in medical care (U)
Illustration	“They’re less involved but they’re still concerned. They’ll ask how the doctor’s appointment was and what we talked about, but in a very respectful manner. They’re not overbearing in the information that they want me to provide to them.” (A young adult)^(p.420)^
Finding	Emerging adult comfort with disclosure (U)
Illustration	“I feel much more comfortable talking to her about pretty much anything; like ‘hey, I’m having a problem with this, and it’s kind of personal and embarrassing, but it’s an issue.’” (A young adult)^(p.420)^
Tremblay *et al.*,^49^ 2020	
Finding	Lack of formal preparation (U)
Illustration	I haven’t really thought, actually I have, I’ve thought about like, oh, what’sgoing to happen when I’m older…because Iknow that you can keep coming here until you’re like 18 or something like that? (15-year-old female, diabetes duration 9 years)^(p.334)^
Finding	Desire for delayed and gradual transition (U)
Illustration	I don’t want to talk about it…. I just turned18, so I don’t want to leave yet. (18-year-old female, diabetes duration 7 years)^(p.334)^
Finding	Attachment to pediatric providers (U)
Illustration	I’ve been with the same doctors for a very long time so, you know, I feel very comfortable with them. (22-year-old male, diabetes duration 12 years)^(p.334)^
Finding	Concern about an impersonal adult care setting (U)
Illustration	Umm, my concern about it is, like, having doctors trying to just, like, dose me up, like I’m saying earlier. And, like, not understanding what’s, like, my history, my background history. (16-year-old female, diabetes duration 2 years)^(p.335)^
Vaillancourt *et al.*,^48^ 2023	
Finding	Minimal or no advice, teachings, or information about the transition to adult care (U)
Illustration	“Yeah my previous pediatric doctor told me that yes, you will be transferred to the adult clinic, that’s all.” (Participant 96, female, age=19 years)^(Suppl 2)^
Finding	Minimal difference between pediatric and adult care (U)
Illustration	“Um it hasn’t been super different. The only thing that’s been kinda different is that I see the nurses less than I did at the children’s. But overall the experience has been the same, like, I feel the same, like, doctor-patient relationship has been pretty much the same at the Adult and the Children’s.” (Participant 18, female, age=19 years)^(Suppl 2)^
Finding	Quick and abrupt change to adult care (U)
Illustration	“Well, as I was saying earlier, it’s really a bit of a laissez-faire. Like really things are very different. At the Children’s (clinic) we are very supported, and then next day it is as if you are left for yourself a little bit. There is no support as such, it’s like “you’re an adult, you are able to take care of things”. It’s not because you’re going from 17 to 18 years old that you’re managing diabetes properly and it’s the same thing as when you were in the Children’s (clinic) but now you have to take responsibility as an adult.” (Participant 100, female, age=18 years)^(Suppl 2)^
Finding	Greater motivation for self-management (U)
Illustration	“Ya, more responsibility I stopped joking, like before I used to sometimes go a couple of days without taking my insulin, now I’m being more serious, being more strict about it, ‘cause I’m starting to drive, like I’m driving it’s dangerous if I don’t take my insulin and I go for a drive, I’m in university, I have to get ready for the exams, I can’t be messing around with my insulin doses, or missing doses either.” (Participant 48, male, age=19 years)^(Suppl 2)^
Finding	The transition to adult care was concurrent with their age and developmental period (U)
Illustration	“I felt like I really just, like, as a person matured more, and like, taking care of myself, it like, really like, coincided with me becoming an adult, especially in my diabetes development, I became more mature, knowing how to handle myself for my own good and just taking more initiatives, so I’ve definitely, ya, I think it’s worked out fine.” (Participant 2, male, age=18 years)^(Suppl 2)^
Finding	Change in their health care provider’s expectations (U)
Illustration	“‥There is certainly no babying going on in adult care. It’s very much “this is your responsibility, it’s nobody else’s” and I agree with them, but it’s, ya it’s more intense, that’s for sure.” (Participant 89, male, age=19 years)^(Suppl 2)^
Finding	Social support (U)
Illustration	“My parents are just really supportive about that, they want me to get back in control and eating healthy and being portioned. We’re starting carb counting but I’m not 1000% confident with that yet. So I’m just started with portions and doing that. My parents are extremely supportive about that and they’re constantly on my case about it.” (Participant 24, male, age=19 years)^(Suppl 2)^
Finding	Health care organization (U)
Illustration	“I have a lot of flexibility for appointments, they reserve a whole schedule space for students, who of course do not want to miss too much school; I have fixed days but I have a whole schedule of space according to what’s convenient. And when I arrive there, it doesn’t take long; not more than 15 minutes.” (Participant 44, female, 19 years)^(Suppl 2)^
Finding	Relationship with health care provider (i.e. getting along well) (U)
Illustration	“Well really, the fact that I got along well with my new doctor, and I have the impression that it is a better match than with my previous doctor.” (Participant 30, female, age=19 years)^(Suppl 2)^
Finding	Timing of preparation about transition (U)
Illustration	“Ya I did feel ready, Dr. “D” really spoke about it quite a lot before I made the switch, I think a year before where we talked about it in our visits. So ya, I was definitely ready.” (Participant 75, female, age=19 years)^(Suppl 2)^
Finding	Change in provider expectations (i.e. greater autonomy and responsibility) (U)
Illustration	“I think it helped me a little bit because when I was at the Children’s I was getting a lot of help from the team and from my parents and when I transitioned over to the adult everything was put on me, so it was a little bit hard at first but it, uh, really made me find that I had to want to take care of my health and make sure that now I was on top of everything and I really had to push myself to be ready to start my insulin pump and stuff like that and to make sure that I was on top of everything that needed to be done, making sure that I did everything myself.” (Participant 14, female, age=19 years)^(Suppl 2)^
Finding	Shared decision-making with their health care team (U)
Illustration	“Well that’s it, ya like participating more during the sessions with the doctor because I really get to see what she’s doing and pay more attention to my numbers and stuff like that.” (Participant 27, female age=19 years)^(Suppl 2)^
Finding	Commencement of insulin pump therapy (U)
Illustration	“Maybe the support I really got at the hospital, especially when I changed to the insulin pump around 14 years of age, like, I really saw the nutritionist again and the nurse and everything, they really supported me at that level so now after I feel ready to manage myself.” (Participant 66, female, age=19 years)^(Suppl 2)^
Finding	Longer duration of illness (U)
Illustration	“I had the chance, well I don’t know if I can call it luck, but having diabetes since I was 17 months old, so I really learned to manage it myself throughout the years, so I am fine with it.” (Participant 66, female, age=19 years)^(Suppl 2)^
Finding	Access to transitional care (U)
Illustration	“I just knew that it was coming, and I think that the fact that it’s like an in-between space, like it’s not completely just adults, it’s like an intermediary, I think that helped too. It’s less scary than being with like grown-up adults.” (Participant 27, female age=19 years)^(Suppl 2)^
Finding	Minimal or lack of advice or information from pediatric health care provider (U)
Illustration	“I’d say that I definitely didn’t understand all of my options and um I feel like it’s made it difficult since leaving, and also like, if I wanted to change my option now from being at a private clinic to going to a hospital, I wouldn’t even know how to go about that. I don’t want to that, I’m just saying, but if I did want to do that, um I don’t know, I just feel like it was the last appointment that we talked about it and by then it’s too late cause I’m not seeing my doctor again. If I was to change doctors I feel like it should be done almost a year in advance. That would, for sure make the process much easier.” (Participant 15, male, age=19 years)^(Suppl 2)^
Finding	Loss of social support (U)
Illustration	“Not very well, I did not feel supported. For example, during the Summer I had a lot of hyperglycemia. And she thought that it was still fine, my blood sugars, when I was often 15 or things like that and I knew that my blood sugars were not nice and there wasn’t much support so that I would be more careful. She would make changes but not changes that would make it better. Also, everything regarding paperwork, like papers to complete for the government for diabetes, she did not want to complete. She said it was useless and that I had to complete them or my family doctor. No (pause) she is not present for me for that, she is more laissez-faire compared to the Children’s.” (Participant 100, female, age=18 years)^(Suppl 2)^
Finding	Health care or clinic organization (U)
Illustration	“That’s what I think sucks, we don’t have much follow-up compared to the Children’s and we don’t have a direct link with the doctor for anything. With Dr “E” we could call as soon as I had a problem and I would send a message and he would answer within a week. With her (adult provider) its only by appointment and in between there is no sign of life, I cannot get the information I would like to have.” (Participant 100, female, age=19 years)^(Suppl 2)^
Finding	Separation of health care services (e.g. nutritionist, ophthalmologist, nurse) (U)
Illustration	“Well for my blood draw appointments, I have to figure out to make them, there is no nurse, there is no nutritionist, for all my nutrition questions, it’s only her (endocrinologist) and three quarters of the questions that I ask, she doesn’t know the answer to.” (Participant 100, female, age=18 years)^(Suppl 2)^
Finding	Change in provider expectations (i.e. greater autonomy and responsibility) (U)
Illustration	I feel like there’s, again, a lot more independent and autonomous, so there was that that was quite a harsh transition because I wasn’t used to doing things on my own. But they didn’t really leave me in the void trying to figure it out on my own. They were still there to help me out. I would say that the autonomous part of it was the hardest.” (Participant 75, female, age=19 years)^(Suppl 2)^
Finding	Change in health care provider and staff (U)
Illustration	“The only thing was maybe the transitioning period because I had seen Dr. “A” from since (I was) 9 years old until almost 18 so I think that was the only hard thing because I was so comfortable with him, so after being with him for so many years it was just the sense of getting comfortable with the new doctor.” (Participant 94, female, age=19 years)^(Suppl 2)^
Finding	Medical appointments (U)
Illustration	“And more appointments be more frequent than every 6 months because every 6 months is not representative of what we live on a day to day basis.” (Participant 100, female, age=18 years)^(Suppl 2)^
Finding	Visit the adult care clinic and meeting the staff prior to their move to adult care (U)
Illustration	“I think maybe if I would have met Dr. “C” maybe before seeing her for the first time, like with Dr. “A” probably would have maybe been a little easier because I would have seen her before so it wasn’t the first time that I see her as my doctor in the appointment, it probably would have been a little easier.” (Participant 94, female, age=19 years)^(Suppl 2)^
Finding	Access to educational information (U)
Illustration	“Ya, and if the process was started earlier and I had a better explanation, like instead of having your doctor give you the explanation maybe something who is trained in explaining the options, it would make more sense? I feel like that would be a more beneficial process.” (Participant 15, male, age=19 years)^(Suppl 2)^
Visentin *et al.*,^51^ 2006	
Finding	Education and dietetic advice was reactive not proactive (U)
Illustration	…young adults who are in their early 20s and they hadn’t actually been re-taught anything about their diabetes so they had no idea what sick days were or what to do. They didn’t know much about ketoacidosis and had…dated ideas, around food and exercise and that sort of thing…. Lots of issues around obviously that they just haven’t been re-educated. They’ve just run off the information that their parents had given them and that seemed very ‘hit and miss’. That seemed to be only if they had problems, that wasn’t very proactive. So, the endocrinologist would refer only if they were a problem. (diabetes nurse educator)^(p.765)^
Finding	The children’s model is different to the adult model (U)
Illustration	The paediatric model is quite different from the adult model of care in that if they don’t come to clinic…we chase them, whereas in the adult field…it is up to them to do the chasing…the adult model is…here is the information, do with it what you will. In the paediatric scene…we don’t…give up on them…they’re babied you know. (Pediatrician)^(p.766)^
Finding	Not all phases of the transition process are being adequately addressed (U)
Illustration	at the moment, there’s no follow-up of whether the transition has worked smoothly or not. (A health professional)^(p.767)^
Walsh *et al.*,^55^ 2018	
Finding	Hardly noticed it (U)
Illustration	“I can’t even remember when it was but like, I don’t remember them saying anything special like…” (a young adult)^(p.787)^
Finding	An inevitable step (U)
Illustration	“I think it was just that, you get old enough, go to the adult one…simple as that” (a young adult)^(p.787)^
Finding	A physical move (U)
Illustration	“I was just moved from… the teenage, or the younger clinic, to the adult one. That was it really” (a young adult)^(p.787)^
Finding	Self-management (U)
Illustration	“I suppose it’s a very manageable part of my life…‥ I don’t see any difference between that and brushing my teeth” (a young adult)^(p.787)^
Finding	Exclusion from adult services (U)
Illustration	“… they kind of don’t encourage us to come” (a parent of a young adult)^(p.787)^
Finding	Young adult’s self-management (U)
Illustration	“I was glad to see her taking responsibility for herself… rather than me hounding her” (a parent of a young adult)^(p.787)^
Finding	Timing of transition (U)
Illustration	“She says now she prefers the young adult unit because they talk to her like a young adult” “she felt she was too old to be in Paeds… But then as a parent preferred it so… you can’t keep everyone happy” (a parent of a young adult)^(p.787)^
Finding	Preparation (U)
Illustration	“you let them know that they’ll be moving on” (a health care professional)^(p.787)^
Finding	Referral (U)
Illustration	“discharge summary from paeds… tells you everything you need to know about them” (a health care professional)^(p.787)^
Finding	Key person (U)
Illustration	“A is the paediatric and the adult nurse” (a health care professional)^(p.787)^
	“You always knew you could pick up the phone and ring them… it’s a nice kind of a safety net to have” (a parent of a young adult)^(p.787)^
Finding	Education (U)
Illustration	“every consultation, alcohol is talked about with every single young adult… same with smoking” (a health care professional)^(p.787)^
Finding	Attendance (U)
Illustration	“You have a 50% did not attend rate” (a health care professional)^(p.787)^
Finding	Approach to transition (U)
Illustration	“It’s very tick-the-box” (a health care professional)^(p.787)^
Finding	Lack of psychology service (U)
Illustration	“You will know medically what is going on with them but from a psychological point of view it is the joint paediatric-adult ANP that helps with that” (a health care professional)^(p.787)^
Finding	Timing (U)
Illustration	“There’s a lot of change in a young person’s life - going to college, leaving home…makes it even more difficult” (a health care professional)^(p.787)^
	“Yeah, probably for me personally… earlier transition would be better… but that may not be the same for everyone” (a young adult)^(p.787)^
Finding	Continuity (U)
Illustration	“I haven’t moved diabetes nurse and I have quite a good relationship with her” (a young adult)^(p.787)^
Finding	Consultation style (U)
Illustration	“Encouragement rather than criticism” (a young adult)^(p.787)^
	“Very approachable; she seemed very dedicated” (a parent of a young adult)^(p.787)^
Finding	Flexibility (U)
Illustration	“Come in any day you have any question or give me a call” (a young adult)^(p.787)^
Finding	Transition structure (U)
Illustration	“Moved on to a teenage service in the pediatric unit… I found it very good” (a young adult)^(p.787)^
Finding	Follow up (U)
Illustration	“Good so to have a sort of similar routine” (a parent of a young adult)^(p.787)^
Finding	Attitude (U)
Illustration	“I know I should look after myself, I’m well aware of the consequences, you know. But I just really don’t care, to tell you the truth.” (a young adult)^(p.787)^
	“He doesn’t give one tuppenny damn about diabetes; he wishes it wasn’t there, he’s kind of angry about it at the moment” (a parent of a young adult)^(p.787)^
Finding	Other priorities (U)
Illustration	“when you’re in college, you don’t want to take a day out to travel to Galway to go to a clinic” (a young adult)^(p.787)^
Finding	Resource limitations (U)
Illustration	“Appointments only twice a year… not enough… that’s the health service and the cutbacks” (a parent of a young adult)^(p.787)^
Finding	Lack of structured education (U)
Illustration	“You can ring for advice, but there’s no actual program of education” (a parent of a young adult)^(p.787)^
Williams *et al.*,^53^ 2021	
Finding	More structured processes (U)
Illustration	“I think we need to start our young people early and let them know and let them get excited about transitioning themselves.” Diabetes educator^(p.114)^
Finding	Shared educational resources (NS)
Illustration	None
Finding	Expanding the role played by primary care physicians (U)
Illustration	“Where it’s easier for them [the patient] to get in to see maybe their [primary care physician], as opposed to coming in to see the [diabetes care] team… that would be a really great way to keep them connected with the health care system.” Paediatrician^(p.114)^
Finding	A dedicated transfer clinic (NS)
Illustration	None

U, unequivocal; C, credible

ANP, advanced nurse practitioner; GP, general practitioner; HCP, health care provider; NS, not supported; PDC, pediatric diabetes clinic; T1D, type 1 diabetes.

## Appendix V: Synthesized findings

**Table:**

Synthesized finding	Categories	Findings	Illustrations
i) Adolescents and young adults with T1D may be anxious and feel unprepared due to being unaware of the process and a lack of preparation; however, others may be confident and ready for their transition to adult services.	Transition preparedness and associated anxiety	Lack of formal preparation (U)	I haven’t really thought, actually I have, I’ve thought about like, oh, what’sgoing to happen when I’m older…because I know that you can keep coming here until you’re like 18 or something like that? (15-year-old female, diabetes duration 9 years)^49^^(p.334)^
		Limited information about the transition (U)	I cannot quite remember, but I do not think I received any information. I received a letter about the next appointment at the adult outpatient clinic. (a young adult)^36^^(p.726)^
		Minimal or no advice, teachings, or information about the transition to adult care (U)	Yeah my previous pediatric doctor told me that yes, you will be transferred to the adult clinic, that’s all. (Participant 96, female, age=19 years)^48^^(Suppl 2)^
		Minimal or lack of advice or information from pediatric health care provider (U)	I’d say that I definitely didn’t understand all of my options and um I feel like it’s made it difficult since leaving, and also like, if I wanted to change my option now from being at a private clinic to going to a hospital, I wouldn’t even know how to go about that. I don’t want to that, I’m just saying, but if I did want to do that, um I don’t know, I just feel like it was the last appointment that we talked about it and by then it’s too late cause I’m not seeing my doctor again. If I was to change doctors I feel like it should be done almost a year in advance. That would, for sure make the process much easier. (Participant 15, male, age=19 years)^48^^(Suppl 2)^
		Nonpurposeful transition (U)	There really wasn’t much discussion about it. Other than where I was going to go. Yeah, I just kind of had to take it from there. [female, age 25]^42^^(p.194)^
		Abrupt transfer between clinics (U)	I’m happy about the way they treated me and that, it [i.e., the transfer] went well, but it felt really abrupt. Overnight, there were completely new people and despite them being very kind there’s like something completely new. Especially when there is such a difference between how the paediatric and the adult clinics work. If the transfer could proceed more slowly, it would not be as abrupt. (Participant 7, a young adult)^37^^(p.4629)^
		Quick and abrupt change to adult care (U)	Well, as I was saying earlier, it’s really a bit of a laissez-faire. Like really things are very different. At the Children’s (clinic) we are very supported, and then next day it is as if you are left for yourself a little bit. There is no support as such, it’s like “you’re an adult, you are able to take care of things”. It’s not because you’re going from 17 to 18 years old that you’re managing diabetes properly and it’s the same thing as when you were in the Children’s (clinic) but now you have to take responsibility as an adult. (Participant 100, female, age=18 years)^48^^(Suppl 2)^
		Desire for delayed and gradual transition (U)	I don’t want to talk about it…. I just turned 18, so I don’t want to leave yet. (18-year-old female, diabetes duration 7 years)^49^^(p.334)^
		Perceived responsibility of paediatric healthcare team (C)	Obviously I don’t know anything about like, if there’s any differences, what they have here [in paediatrics] and there [in adult care] so it would be good to know, understand what it’s like. (Participant 36, an adolescent)^43^^(p.7)^
		Change in their health care provider’s expectations (U)	‥.There is certainly no babying going on in adult care. It’s very much “this is your responsibility, it’s nobody else’s’ and I agree with them, but it’s, ya it’s more intense, that’s for sure. (Participant 89, male, age=19 years)^48^^(Suppl 2)^
	Unawareness of the transition process	Transition unawareness (U)	I don’t think it was something we talked about. I think it was perhaps the last consultation or the one before that, they mentioned, that it was time for me to move. There wasn’t been any communication about it. I don’t think, there was. (U10, an adolescent)^58^^(p.54)^
		Hardly noticed it (U)	I can’t even remember when it was but like, I don’t remember them saying anything special like… (a young adult)^55^^(p.787)^
		An inevitable step (U)	I think it was just that, you get old enough, go to the adult one…simple as that. (a young adult)^55^^(p.787)^
		A physical move (U)	I was just moved from… the teenage, or the younger clinic, to the adult one. That was it really. (a young adult)^55^^(p.787)^
		Fatality, passivity and waiting (U)	It’s inevitable. (a young adult)^57^^(p.414)^
	Confidence to transition	The transition to adult care was concurrent with their age and developmental period (U)	I felt like I really just, like, as a person matured more, and like, taking care of myself, it like, really like, coincided with me becoming an adult, especially in my diabetes development, I became more mature, knowing how to handle myself for my own good and just taking more initiatives, so I’ve definitely, ya, I think it’s worked out fine. (Participant 2, male, age=18 years)^48^^(Suppl 2)^
		Minimal difference between pediatric and adult care (U)	Um it hasn’t been super different. The only thing that’s been kinda different is that I see the nurses less than I did at the children’s. But overall the experience has been the same, like, I feel the same, like, doctor-patient relationship has been pretty much the same at the Adult and the Children. (Participant 18, female, age=19 years)^48^^(Suppl 2)^
		Timing (U)	Yeah, probably for me personally… earlier transition would be better… but that may not be the same for everyone. (a young adult)^55^^(p.787)^
		Security with the familiar (U)	I got it [i.e., the disease] at an early age. I suppose that’s better than later. I was already managing my diabetes when I got to that age [i.e., the transition to adulthood] for that reason. It was not a big deal—it was already part of my daily life. (Participant 3, a young adult)^37^^(p.4628)^
		Age of diagnosis affecting comfort with self-management (U)	Of course, it’s different maybe for other kids if you’ve been diagnosed when you were like 14 … and you’re not used to living with it whereas if you’re diagnosed at 5 like I was and it’s part of you, so for me there’s definitely like less that I have to learn. (Participant 13, an adolescent)^43^^(p.6)^
		Longer duration of illness (U)	I had the chance, well I don’t know if I can call it luck, but having diabetes since I was 17 months old, so I really learned to manage it myself throughout the years, so I am fine with it. (Participant 66, female, age=19 years)^48^^(Suppl 2)^
		Self-management (U)	I suppose it’s a very manageable part of my life…‥ I don’t see any difference between that and brushing my teeth. (a young adult)^55^**^(p.787)^**
		Perceived self-responsibility (U)	I have to be more responsible, more consistent, doing more blood sugars, more testing, so, basically managing […] my diabetes a bit better because I’m gonna be alone. (Participant 53, an adolescent)^43^^(p.7)^
		Greater motivation for self-management (U)	Ya, more responsibility I stopped joking, like before I used to sometimes go a couple of days without taking my insulin, now I’m being more serious, being more strict about it, ‘cause I’m starting to drive, like I’m driving it’s dangerous if I don’t take my insulin and I go for a drive, I’m in university, I have to get ready for the exams, I can’t be messing around with my insulin doses, or missing doses either. (Participant 48, male, age=19 years)^48^^(Suppl 2)^
		Developmentally appropriate interaction (U)	I was finally being treated like the adult I should have been. At the age of 17 I feel I was too independent to have [my diabetes center] ‘holding’ my hand through diabetes care. I think once a human matures they no longer need their doctors to treat them like they are 10 years old. (20-year-old male)^44^^(p.350)^
		Emerging adult comfort with disclosure (U)	I feel much more comfortable talking to her about pretty much anything; like ‘hey, I’m having a problem with this, and it’s kind of personal and embarrassing, but it’s an issue.’ (a young adult)^56^^(p.15)^
ii) Adolescents with T1D face many barriers as they transition from pediatric to adult care, mainly due to challenges of navigating changes in health systems or the burden of living with T1D.	Health system challenges	Attachment to pediatric providers (U)	I’ve been with the same doctors for a very long time so, you know, I feel very comfortable with them. (22-year-old male, diabetes duration 12 years)^49^^(p.334)^
		Loss of bond with paediatric team (U)	Umm well personally, I’ve known my doctor since I was like 5. Umm I feel like I have a bond with them and I feel like going to a new doctor I won’t be as comfortable talking about some of the things that could be important that I might not necessarily think as that important but could turn out to be serious later. (Participant 5, Focus Group 2, a young adult)^50^^(p.5)^
		Leaving paediatric services (U)	In children’s… once every 4 months you’d get all the specialists come up from (city) hospital, which was really good. And you’d go along and they’d do your HbA1c and they would talk to you about it and you know, you felt like they were… at the top of the field kind of thing. But once you turn eighteen… all that kind of stuff, it’s like it just stops. (male, 27 years)^13^^(p.1958)^
		Attachment to paediatric healthcare team (U)	I’m actually kind of sad that I’m leaving the team, they were so great. (Participant 12, an adolescent)^43^^(p.6)^
		Loss and gain in provider relationships across the transition (U)	I think the hardest thing in health care… is that you build the relationship with the person…. That is why I didn’t want to leave [pediatrics]. Like I felt like they got me and like they totally knew what I was about, and that is one of the hardest things… about adult care is rebuilding that relationship. (female, 25 years old, transitioned at age 23)^45^^(p.43)^
		Loss of social support (U)	Not very well, I did not feel supported. For example, during the Summer I had a lot of hyperglycemia. And she thought that it was still fine, my blood sugars, when I was often 15 or things like that and I knew that my blood sugars were not nice and there wasn’t much support so that I would be more careful. She would make changes but not changes that would make it better. Also, everything regarding paperwork, like papers to complete for the government for diabetes, she did not want to complete. She said it was useless and that I had to complete them or my family doctor. No (pause) she is not present for me for that, she is more laissez-faire compared to the Children’s. (Participant 100, female, age=18 years)^48^^(Suppl 2)^
		A crucial change in relationship with health care providers (U)	What’s relevant for me and for you is my diabetes and my insulin pump. If I have any other questions and questions on my blood sugar. That’s what relevant. In the pediatric ward, it was more like; what are you doing in your vacation and so on. There is less of that here, and that’s fine by me. (U1, an adolescent)^58^^(p.56)^
		Change in provider expectations (i.e. greater autonomy and responsibility) (U)	I feel like there’s, again, a lot more independent and autonomous, so there was that that was quite a harsh transition because I wasn’t used to doing things on my own. But they didn’t really leave me in the void trying to figure it out on my own. They were still there to help me out. I would say that the autonomous part of it was the hardest. (Participant 75, female, age=19 years)^48^^(Suppl 2)^
		Concern about an impersonal adult care setting (U)	Umm, my concern about it is, like, having doctors trying to just, like, dose me up, like I’m saying earlier. And, like, not understanding what’s, like, my history, my background history. (16-year-old female, diabetes duration 2 years)^49^^(p.335)^
		Fear of not having bond with adult team (U)	For me, definitely for the doctor, I want somebody who is social and isn’t there to just be a doctor. I want somebody there who wants to learn about me, to kinda get to know me, kinda be like a friend, so it’s not such a blatant appointment about this needs to be better, this needs to change, this – so it’s not just all about diabetes. (Participant 1, Focus Group 1)^50^^(p.6)^
		Worry about future relationship with adult healthcare team (U)	My concern mostly would be that [in adult care] we would lose a little bit of the personal connection with the doctors. [In paediatrics] I feel like it’s a lot more, it’s warm, and it’s very friendly, so … I’m scared of it being very cold. (Participant 75, an adolescent)^43^^(p.6)^
		What teenagers project when they go to the care for adults (U)	There won’t be that little extra. (a young adult)^57^^(p.416)^
		Unexpected differences between pediatric and adult health care systems (U)	The most shocking thing was coming in the first time, and it wasn’t the standard procedure I was used to. Yeah, it’s just something you have to kind of pick up […]. It was just very different. [female, age 25]^42^^(p.194)^
		Transition from a frequent, thorough and personal follow-up to a less comprehensive and less personal follow-up (U)	Yes, because I feel like the doctor is just measuring you, and then you leave again. However, the disease is about so much more than taking insulin. The disease affects you mentally. Therefore, I think the focus on how you are really doing is less evident in the adult outpatient clinic. At the paediatric outpatient clinic, they asked me how I felt and things like that. (a young adult)^36^^(p.726)^
		Access to health professionals (U)	It seems a lot more like a general doctor’s visit now days, which makes it, you know it’s harder to get in to see them. (female, 27 years)^13^^(p.1958)^
		Fragmentation and lack of coordination (U)	I’m good with my diabetes but I don’t like sitting in a room for 4 hours waiting for a doctor, going in and seeing him for 5 minutes and then leaving. There’s not really any point turning up. (female, 23 years)^13^^(p.1958)^
		Limited expectations of how the health care services were organized (U)	I did not feel I had any other choice really. Because at the GP you can get check-ups more often. I was told that there would not be as many follow-ups after the transition to the adult outpatient clinic, so I should get a GP where I could get my mean blood glucose checked more often. (a young adult)^36^^(p.727)^
		Health provider/health system challenges (U)	Jeremy: I called for an appointment to see a doctor…They said that they charged every time you go to an appointment… Eventually, we had to go to the emergency room to get insulin. And they were like, “So why didn’t you go to a doctor [at County]?” And I told them that they wanted to charge me. And they said, no, that is not true, that it is free. (Male, a young adult)^54^^(p.1618)^
		Standardized treatment despite individual needs (C)	There was a great focus on everything that was regarded as positive at the paediatric clinic. But at the adult clinic, it was more like “Your HbA1c outcomes are bad and you are doing this and that wrong”. It led to me frequently leaving the clinic angry. (Participant 6, a young adult)^37^^(p.4630)^
		Age-appropriate services (U)	I personally don’t find (adult diabetes clinics) that useful… it’s really horrible to sit there and watch all the complications they have and what they’re going through. For me, who’s only 26, like that’s quite a big jump in age difference. There was no one my age there. It was all elderly people and it was always late. …. there doesn’t seem to be anything catering for my age group. (female, 26 years)^13^^(p.1959)^
		Care organization (C)	You have to go with the flow.^57^^(p.416)^
		Health care or clinic organization (U)	That’s what I think sucks, we don’t have much follow-up compared to the Children’s and we don’t have a direct link with the doctor for anything. With Dr “E” we could call as soon as I had a problem and I would send a message and he would answer within a week. With her (adult provider) its only by appointment and in between there is no sign of life, I cannot get the information I would like to have. (Participant 100, female, age=19 years)^48^^(Suppl 2)^
		Separation of health care services (e.g. nutritionist, ophthalmologist, nurse) (U)	Well for my blood draw appointments, I have to figure out to make them, there is no nurse, there is no nutritionist, for all my nutrition questions, it’s only her (endocrinologist) and three quarters of the questions that I ask, she doesn’t know the answer to. (Participant 100, female, age=18 years)^48^^(Suppl 2)^
		Change in health care provider and staff (U)	The only thing was maybe the transitioning period because I had seen Dr. “A” from since (I was) 9 years old until almost 18 so I think that was the only hard thing because I was so comfortable with him, so after being with him for so many years it was just the sense of getting comfortable with the new doctor. (Participant 94, female, age=19 years)^48^^(Suppl 2)^
		Challenges of navigating the healthcare system (U)	Young adults do not know what they are doing when they leave paediatric care. Many essential aspects of diabetes care, such as navigating finding a new doctor, getting prescriptions and dealing with insurance are complete mysteries. (25 years old, male)^46^^(p.697)^
	Burden of living with T1D	Stigma of type 1 diabetes (U)	I’ve gotten kicked out of restaurants before … I was very embarrassed at the time. I’m not doing anything wrong, but I can’t really explain because I’m being pushed out of a restaurant. And it wasn’t really worth it at that point to be like, ‘No, like I’m a type 1 diabetic.’ (Participant 14, Focus Group 3, a young adult)^50^^(p.5)^
		Disclosure of diabetes diagnosis (U)	There’s another diabetic at my school… on a pump… He was just typing, this was during one of my… mock exams and the teachers were like, ‘…Why are you on your phone? I thought you handed in your phone?’ And he was like, ‘I’m diabetic…’ Obviously you could tell that he wasn’t comfortable with the whole class knowing. (Participant 19, Focus Group 5, a young adult)^50^^(p.5)^
		Confusion with type 2 diabetes (U)	When they bring up diabetes, they don’t know there is a difference between type 1 and type 2. It’s more just there’s diabetes in general. It can be a little bit frustrating sometimes that stigma around type 2 diabetes. (Participant 12, Focus Group 3, a young adult)^50^^(p.5)^
		Resilience and self-advocacy (U)	It gets annoying cuz I’ve gotten it so much, but I try not to umm be like annoyed with people for being curious. There’s nothing wrong with being curious. But then also like I’ve gotten my explanation talk down to 30 seconds, you know, for what diabetes is. (Participant 18, Focus Group 5, a young adult)^50^^(p.5)^
		Self-care takes work and time (U)	Whatever you are doing, you have an extra hurdle… Whenever I was trying out for soccer teams, it would be such a hassle if I had to deal with my sugars or something. It would be such a setback. It’s a little hard to deal with. It’s annoying… It’s something else that you have to stress about. It’s something else that you have to deal with. Whatever someone else has to do, you have to do more. (Participant 15, Focus Group 4, a young adult)^50^^(p.6)^
		Unpredictability and restrictiveness (U)	You always have to be extra prepared… For example, you’re on the road, there’s no stops, you can’t just like stop and get a pop if you go low or like on an airplane you can’t really, you can, but like… (Participant 22, Focus Group 5, a young adult)^50^^(p.6)^
		Emotional burden (U)	I think people need to be more aware of mental health issues with diabetics as well cuz I found that every single friend… I do know who has diabetes… a lot of them have like depression, a lot of them have anxiety, a lot of them have certain things that they deal with… I feel that the clinic should talk more about that. (Participant 17, Focus Group 4, a young adult)^50^^(p.6)^
		Vulnerability in the college years (U)	All throughout college, the biggest thing I struggled with is balancing my health care and social life. [female, age 23]^42^^(p.194)^
		Adaption to new diabetes care responsibilities (U)	I will be overwhelmed, you can say that, cause sometimes when I’m going out, I usually call my mom to say, because she has the chart at home, so I will always tell her what, how much should I do, but the fact that in 3 months I will be 18 so everything’s on me …. It’s gonna be overwhelming at first but not that much if I get the hang of it. (Participant 48, an adolescent)^43^^(p.6)^
		Developmental challenges (U)	Karen: I do want to [get my HbA1c] below seven because I have had it high this past year. Well, this past month, just because of nursing [school], I was just so caught up, and I was disregarding my diabetes… It’s like out of balance, I guess. It’s just because I was so busy with school. It’s just the pressure from school. (Male, a young adult)^54^^(p.1619)^
		Psychosocial challenges (U)	Roberto: If you go [to the clinic] and you say, I have just a little bit of insulin – because there’s a lot of people waiting to be seen by a doctor – they’re like, then you can come back tomorrow… [They say] come back tomorrow, and it’s going to be faster. And I was like, but I have to go to work, I have to support my family. (Male, a young adult)^54^^(p.1621)^
		Life-changing disease onset (U)	In the beginning, I think I found it a little difficult to take it all in and I did not think about the fact that it would be there for ever and permanently. Later, I was quite sad. (Participant 7, a young adult)^37^^(p.4625)^
		Not always straight forward (U)	I can’t remember getting any information about how this [i.e., the disease] can limit us beyond the clinical picture—how it affects one’s well-being and what kind of everyday life limitations there can be. (Participant 9, a young adult)^37^^(p.4626)^
		Hopeful and challenging future (U)	I believe that the disadvantages for me have been most substantial when I’ve been thinking about what I want to do in the future. I wanted to become a police officer once, but I was held back due to living with diabetes, and that was quite a great loss. (Participant 9, a young adult)^37^^(p.4626)^
		Not like everyone else (U)	I perceive myself as more ill when I’m supposed to pick up a cannula, adjust the pen, and do injections in my stomach. I experience myself as sicker. I also believe it to be harder to do this among others, somehow. (Participant 7, a young adult)^37^^(p.4626)^
		Ambivalence regarding openness (U)	It took a while for my friends in upper secondary school to even know it [i.e., that I was living with T1D]. I didn’t want it to define me anymore, and this was a chance to be “normal” again. (Participant 8, a young adult)^37^^(p.4627)^
		Relationship with other young people (U)	“I don’t want diabetes between me and my friends. (a young adult)^57^^(p.416)^
		Efforts to make peace with oneself (U)	I’ve never come to that [i.e., acceptance] since I didn’t choose to live with it. But anyhow, I’ve arrived at a certain point where I can kind of handle it. That was after my nursing education and it was around 17 to 18 years later [i.e., after the disease onset]. I manage, but I believe I will probably never accept it. (Participant 6, a young adult)^37^^(p.4627)^
		Early responsibility for self- management (U)	I believe it [the responsibility for diabetes self- management] has slowly gone down that path all along. That’s exactly it, there’s more and more responsibility. And here and now, all the responsibility is on me. (Participant 8, a young adult)^37^^(p.4628)^
		Mental health needs of teens with T1D (U)	Doctors on your case when you are trying your best makes it hard to keep trying. I started giving myself a ‘burnout day’ every so often where I did not pay attention to diabetes so that I could feel ‘normal’. (28 years old, female)^46^^(p.697)^
		Managing day-to-day life with T1D (U)	Hormones, activities and diet change during this time. These all account for variation in glucose regulation. (27 years old, female)^46^^(p.698)^
		Shifting of diabetes management from parent to child (U)	Not back off cause that’s [kind of] harsh but like a little bit back off … I feel like even if I make mistakes like I’ll learn from them more than if my parents are always [kind of] bothering me about it … [I kind of] need to do that by myself. (Participant 18, an adolescent)^43^^(p.5)^
		Other priorities (U)	When you’re in college, you don’t want to take a day out to travel to Galway to go to a clinic. (a young adult)^55^^(p.787)^
		Parental anxiety (U)	They get a real kick out of it, they dramatize because they don’t know. (a young adult)^57^^(p.416)^
		Attitude (U)	I know I should look after myself, I’m well aware of the consequences, you know. But I just really don’t care, to tell you the truth. (a young adult)^55^^(p.787)^
iii) A successful transition to adult care for adolescents and young adults with T1D depends on activities to support feeling prepared for the transition, having support networks with a stronger emphasis on peer support, and a structured and coordinated transition between pediatric and adult health care.	Preparation of adolescents and young adults for transition to adult health care	Specific transition preparation (U)	A longer heads up maybe because I feel everyone was just telling you in the last visit to, ‘Hey, you pick a doctor.’ Maybe 5 visits earlier. Making sure you know it’s coming. (Participant 9, Focus Group 2, a young adult)^50^^(p.5)^
		Education (U)	There doesn’t sort of seem to be a lot of help… for the people who are my age, which is probably, in my eyes, which is where you need to be starting to really educate ‘cause it’s now that I’m really listening, (laughs) ‘cause as a teenager they tell you lots of things that if you’re in that state of mind where you don’t take it on board. Whereas now, I take a lot more on board so it’s (laughs) probably more helpful now (female, 26 years)^13^^(p.1960)^
		Transition and diabetes education and preparation (U)	…and I know at the Children’s they were like you need to learn to do carb counting but my Mom did everything for me. She still helps me, but I think now, having an educational class to make sure that us as 17-year-olds actually do know how to manage our diabetes on our own. (Group 2, an adolescent)^47^^(p.4)^
		Early independence to ease transition (U)	Review the stuff that you teach diabetics at the beginning. I learned what caused diabetes before 3rd grade. Now I’m 26 and struggling to remember some of the basics. Help me to manage diabetes in all situations, which my endocrinologists did. This has given me the ability to manage the unexpected moments throughout college and my young adult life. (25 years old, female)^46^^(p.698)^
		Timing of preparation about transition (U)	Ya I did feel ready, Dr. “D” really spoke about it quite a lot before I made the switch, I think a year before where we talked about it in our visits. So ya, I was definitely ready. (Participant 75, female, age=19 years)^48^^(Suppl 2)^
		Commencement of insulin pump therapy (U)	Maybe the support I really got at the hospital, especially when I changed to the insulin pump around 14 years of age, like, I really saw the nutritionist again and the nurse and everything, they really supported me at that level so now after I feel ready to manage myself. (Participant 66, female, age=19 years)^48^^(Suppl 2)^
		Access to educational information (U)	Ya, and if the process was started earlier and I had a better explanation, like instead of having your doctor give you the explanation maybe something who is trained in explaining the options, it would make more sense? I feel like that would be a more beneficial process. (Participant 15, male, age=19 years)^48^^(Suppl 2)^
		Increased level of personal responsibility and diabetes management skills (U)	When my blood sugar is very unstable, we analyze ‘what did I do that day’, and ‘what do you think about the fluctuation right there’ (…) ‘okay, everyday about 7.30 p.m. you are high [in blood sugar level], it can be explained by dinner. Then maybe you should take some more insulin earlier’… We are looking for patterns. (A young adult)^38^^(p.131)^
		Early transition preparation (U)	[Preparation for transition could have been improved if I]…could have slowly been doing things on my own, that way I would already be used to going to the doctors by myself and telling him what was going on. (19-year-old female)^44^^(p.350)^
	Support of adolescents and young adults for transition to adult health care	Peer support (U)	It’s never nice going through something all by yourself. Sure, your parents are there, and they love you, but they don’t know what’s going on. They don’t know how to make you feel better. But somebody who’s going through the same thing knows exactly how you’re feeling. (Participant 1, Focus Group 1, a young adult)^50^^(p.5)^
		Near peer support (U)	I’d say it might have been helpful to have some sort of I dunno phone number for questions or something or maybe even just I dunno some sort of contact with somebody who has done this before and kind-of knows how it went. (Participant 21, Focus Group 5, a young adult)^50^^(p.5)^
		Social and peer support (U)	Just getting together with other people my own age that have diabetes and seeing how they deal with it and having a gathering where you can get information and still have a good time…. partners as well… so that they could also learn more about managing it (female, 22 years)^13^^(p.1960)^
		Social and peer support (U)	I feel like ya know and I think that ya know, it is helpful to be able to talk to someone that at least understands what you are going through. (Group 3, a young adult)^47^^(p.5)^
		Parents empowering autonomy (U)	Yeah, my parents have slowly started stepping back. So, like now I’m monitoring when I’m low on reservoirs or site changes or anything. So, it’s kinda nice, but it’s also like kinda hard to keep track of. It’s slowly piling up. Like everything you have to keep track of. But they’re taking it slow. And they’re slowly letting me take over responsibilities, which is kinda nice. (Participant 11, Focus Group 3, a young adult)^50^^(p.5)^
		Parental involvement in medical care (U)	They’re less involved but they’re still concerned. They’ll ask how the doctor’s appointment was and what we talked about, but in a very respectful manner. They’re not overbearing in the information that they want me to provide to them. (a young adult)^56^^(p.420)^
		Separation from family members but still in need of support (U)	I know some people with T1D and when I meet them, it’s having somebody with the same disease as yourself. Somehow, you become different [when living with the disease], but when you meet these people, you’re some kind of “normal”. I see people who I can speak to. (Participant 4, a young adult)^37^^(p.4629)^
		Social support (U)	My parents are just really supportive about that, they want me to get back in control and eating healthy and being portioned. We’re starting carb counting but I’m not 1000% confident with that yet. So I’m just started with portions and doing that. My parents are extremely supportive about that and they’re constantly on my case about it. (Participant 24, male, age=19 years)^48^^(Suppl 2)^
		Practical and emotional support (U)	We read off my insulin pump and so on. I was not at the hospital, but we just talked on the phone (…) It worked out very well that you were always in contact with someone, so you did not have to deal with it [measure blood sugar levels] by yourself. (A young adult)^38^^(p.131)^
		Importance of support from key players (parents, peers and providers) (U)	Me doing everything early on and having supportive parents really helped me to be independent. (31 years old, female)^46^^(p.697)^
		Support for autonomy (U)	She gives me more options about how to treat things. I have more input. (A young adult)^56^^(p.420)^
		Change in provider expectations (i.e. greater autonomy and responsibility) (U)	I think it helped me a little bit because when I was at the Children’s I was getting a lot of help from the team and from my parents and when I transitioned over to the adult everything was put on me, so it was a little bit hard at first but it, uh, really made me find that I had to want to take care of my health and make sure that now I was on top of everything and I really had to push myself to be ready to start my insulin pump and stuff like that and to make sure that I was on top of everything that needed to be done, making sure that I did everything myself. (Participant 14, female, age=19 years)^48^^(Suppl 2)^
		Family and hospital (C)	Helping the little ones make them feel less alone (a young adult)^57^^(p.415)^
		Social/family support and building a “safety net” (U)	I lost the constant support and attention from my parents that I had always had. I still had support from my parents, friends, and doctor, but overall I had to learn to handle my diabetes independently and take more responsibility for myself and my health, and that was very difficult. (21-year-old female)^44^^(p.350)^
	Health care provision improvement	Having choices in the transition experience (U)	Different people learn differently, like videos would help some people, having the information in front of you would help others. Being able to email that doctor—like having different options—so it’s not just everybody’s going down this one path. (Participant 1, Focus Group 1, a young adult)^50^^(p.5)^
		Improving provider approaches to transition (U)	I feel it might have been helpful to either have some sort of recommendation in place in terms of who I might want to see based on my history and medical details. I guess even just communicating with someone on the adult team to kind of bridge the transition beforehand. It doesn’t even necessarily have to be a physical meeting of everyone, just kind of communicating more. (female, 27 years old, transitioned at age 18)^45^^(p.44)^
		Patient wish list for improving the transition process (U)	If I could make a recommendation to [pediatric providers] regarding pediatric care it would be, even if they only have one adult provider that they recommend, have somebody that you recommend […] I wish that I had received that from my pediatric care […] just something as simple as that would have made a world of difference. [male, age 27]^42^^(p.195)^
		Transition structure (U)	Moved on to a teenage service in the pediatric unit… I found it very good. (a young adult)^55^^(p.787)^
		Access to transitional care (U)	I just knew that it was coming, and I think that the fact that it’s like an in-between space, like it’s not completely just adults, it’s like an intermediary, I think that helped too. It’s less scary than being with like grown-up adults. (Participant 27, female age=19 years)^48^^(Suppl 2)^
		Accessing up-to-date clinical information (U)	I’d like to have more specialists so that in the country you can get more of the newer things that come out. … it would be good to be able to go to a specialist and talk about the options that are, like insulin pumps and all that kind of stuff, but it’s just really not an option here (male, 27 years)^13^^(p.1959)^
		Communication technology (U)	I think a text message or an email would sort of remind you to keep on track. (Group 1, an adolescent)^47^^(p.4)^
		Coordinating care (U)	Having appointments where both the pediatric and adult care professionals would be there to meet and discuss my treatment. (21-year-old male)^44^^(p.351)^
		Health care organization (U)	I have a lot of flexibility for appointments, they reserve a whole schedule space for students, who of course do not want to miss too much school; I have fixed days but I have a whole schedule of space according to what’s convenient. And when I arrive there, it doesn’t take long; not more than 15 minutes. (Participant 44, female, age=19 years)^48^^(Suppl 2)^
		Medical appointments (U)	And more appointments be more frequent than every 6 months because every 6 months is not representative of what we live on a day to day basis. (Participant 100, female, age=18 years)^48^^(Suppl 2)^
		Meeting adult provider before transition (U)	Maybe like for [age] 17–19 you’re meeting with your children’s doctor and the doctor you’re going to after, so they can talk about what’s best for you and so they can learn about you together so that your children’s doctor can talk to your new doctor about how he’s found or she’s found best ways to help you. (Participant 1, Focus Group 1, a young adult)^50^^(p.5)^
		Visit the adult care clinic and meeting the staff prior to their move to adult care (U)	I think maybe if I would have met Dr. “C” maybe before seeing her for the first time, like with Dr. “A” probably would have maybe been a little easier because I would have seen her before so it wasn’t the first time that I see her as my doctor in the appointment, it probably would have been a little easier. (Participant 94, female, age=19 years)^48^^(Suppl 2)^
		Continuity in the relationship with the diabetes nurse (U)	She [the diabetes nurse] has been with me from the beginning, so she knows that I can manage diabetes… but she also knows that I sometimes fail to manage it. But she knows what can get me on the right track. (Sophie, female, a young adult).^38^^(p.130)^
		Continuity (U)	I haven’t moved diabetes nurse and I have quite a good relationship with her. (a young adult)^55^^(p.787)^
		Flexibility (U)	Come in any day you have any question or give me a call. (a young adult)^55^^(p.787)^
		The importance of being seen as a whole person (U)	Now, I feel in a way that talking about diabetes is a personal thing and how I have handled it or are handling it is personal. It would be easier to talk about it with people I have met before than with total strangers. (A young adult)^36^^(p.727)^
		HCP consistency (U)	The fact that she recognizes me when I walk into the clinic, or remembers something I said in my last appointment, is nice to know that she actually knows who I am as a human being, and I think that’s really important. (A young adult)^56^^(p.420)^
		HCP interaction style (U)	She definitely does listen to me; it’s refreshing to have a physican who cares that much. (A young adult)^56^^(p.420)^
		Partnership without shared decision-making (U)	Usually you agree with the doctors. They know what is best, besides yourself, of course. Usually you agree. I have never tried to say no – I don’t think that’s a good idea. You make sure, you agree and usually that’s what you do (U1, an adolescent)^58^^(p.57)^
		Importance of trusting, caring relations (U)	We, who have T1D, come here regularly and the visits are not that personal, so maybe you [i.e., HCPs] could spend a little more time with us. Who we are, what kind of life we are living, and what kind of support we need, and so on. I’d desired that, not simply being a name on a paper. (Participant 1, a young adult)^37^^(p.4630)^
		Shared decision making with their health care team (U)	Well that’s it, ya like participating more during the sessions with the doctor because I really get to see what she’s doing and pay more attention to my numbers and stuff like that. (Participant 27, female age=19 years)^48^^(Suppl 2)^
		A personal patient–provider relationship (U)	My doctor sometimes says I should try scuba diving, he has tried it a lot, and then we talk about many different places. I went to Cuba and then he said, ‘I really want to go there’. We talk about personal things (…) I think it is very nice and it makes the check-ups less serious and creates a homelier atmosphere. (Anna, female, a young adult).^38^^(p.130)^
		Relationship with health care provider (i.e. getting along well) (U)	Well really, the fact that I got along well with my new doctor, and I have the impression that it is a better match than with my previous doctor. (Participant 30, female, age=19 years)^48^^(Suppl 2)^
		Partners in care versus on one’s own (U)	It’s [adult care] less of the dictating and more of the collaboration, which has been good…. So, it’s more of a conversation rather than a, “this is what you have to do” type of thing. So that’s been helpful. (male, 24 years old, transitioned at age 18)^45^^(p.43)^
		Consultation style (U)	Encouragement rather than criticism (a young adult)^55^^(p.787)^
iv) Parents can struggle with their child’s transition where the parents’ roles and responsibilities are refined by their children as they approach adulthood, and parents may need support to address their new role during transition.	Parental perceived challenges of transition	Parental experiences of transition (U)	I kind of had a period of ‘what now’? You know, like I mean it was never my disease to deal with [but] now what’s my role? And figuring out you know, where I fit. (P3, a parent)^41^^(p.3)^
		Renegotiating parent–child roles, responsibilities and relationships (U)	You want to give them enough rope so that they can learn things for themselves, but not enough to hang themselves with (P6, a parent).^41^^(p.4)^
		New and evolving fears (U)	I think now the biggest [fear] is that, all through the years you have some sort of control…and now I can’t tell you what his last A1C was. That’s all information that he keeps to himself…That was a big shift, to kind of relinquish control when I had so much control. (P3, a parent)^41^^(p.4)^
		The ambiguous positioning of mothers (U)	I don’t know what’s expected of me anymore and I don’t know what to expect from them…he’s an adult and they keep saying that…but for me he’s still my son and he’s still got diabetes and he still needs help and guidance. (1-M137, a mother)^52^^(p.997)^
		Exclusion from adult services (U)	… they kind of don’t encourage us to come. (a parent of a young adult)^55^^(p.787)^
		Timing of transition (U)	She says now she prefers the young adult unit because they talk to her like a young adult … she felt she was too old to be in Paeds… But then as a parent preferred it so… you can’t keep everyone happy (a parent of a young adult)^55^^(p.787)^
		Timing of transfer (U)	He can stay here [in pediatric care] forever. For my money, he doesn’t have to go anywhere. I’d like to see him be a bit older. (mother to 16-year-old male)^44^^(p.350)^
		Lack of structured education (U)	You can ring for advice, but there’s no actual program of education. (A parent of a young adult)^55^^(p.787)^
		Resource limitations (U)	Appointments only twice a year… not enough… that’s the health service and the cutbacks. (A parent of a young adult)^55^^(p.787)^
		Attitude (U)	He doesn’t give one tuppenny damn about diabetes; he wishes it wasn’t there, he’s kind of angry about it at the moment. (A parent of a young adult)^55^^(p.787)^
	Parental perceived facilitators of transition	The transition from child to adult services (C)	I’m involved when something is out of the ordinary, if it’s the ordinary she just gets on with it, if she thinks something is a bit different to what she’s expecting then we’ll discuss things with her. (4-M50, a mother)^52^^(p.997)^
		Young adult’s self-management (U)	I was glad to see her taking responsibility for herself… rather than me hounding her. (a parent of a young adult)^55^^(p.787)^
		Early transition preparation (U)	Well, she’s mentioned how when she goes away to college, how is she going to keep track of her numbers at night, and how is she going to manage that. So, she’s already anticipating that, which I think is great. (mother of a 16-year-old female)^44^^(p.350)^
		Social/family support and Building a “safety net” (U)	I think the challenge at college is just going to be not having the safety net and the transition with mom and dad, and not having the friends that she has known and her friends who know her diabetes….Mom and dad aren’t going to be there every day saying, ‘What’s your blood sugar? What’s your bolus?’ And I just think it’s going to be a huge, huge, huge shift in responsibility when she realizes. (mother of 16-year-old female)^44^^(p.350)^
		Consultation style (U)	Very approachable; she seemed very dedicated. (A parent of a young adult)^55^^(p.787)^
		Follow up (U)	Good so to have a sort of similar routine. (A parent of a young adult)^55^^(p.787)^
		Key person (U)	You always knew you could pick up the phone and ring them… it’s a nice kind of a safety net to have. (A parent of a young adult)^55^^(p.787)^
v) Health care providers can be uncertain of the best transition strategy and tend to suggest many activities to improve transition, mainly focusing on coordination between pediatric and adult health care.	HCPs’ perceived challenges of transition	Uncertainty of appropriate strategies (U)	They don’t listen to this just now… they don’t think it is particularly important or interesting I would like to find other ways to meet‥ perhaps it’s not the right time. (I-A, a health care professional)^40^^(p.4)^
		Education and dietetic advice was reactive not proactive (U)	…young adults who are in their early 20s and they hadn’t actually been re-taught anything about their diabetes so they had no idea what sick days were or what to do. They didn’t know much about ketoacidosis and had…dated ideas, around food and exercise and that sort of thing…. Lots of issues around obviously that they just haven’t been re-educated. They’ve just run off the information that their parents had given them and that seemed very ‘hit and miss’. That seemed to be only if they had problems, that wasn’t very proactive. So, the endocrinologist would refer only if they were a problem. (diabetes nurse educator)^51^^(p.765)^
		Not all phases of the transition process are being adequately addressed (U)	At the moment, there’s no follow-up of whether the transition has worked smoothly or not. (A health professional)^51^^(p.767)^
		Deciding time for transfer (U)	I think that there are quite a lot who almost beg and pray to stay and they bring up the question themselves of when they have to leave. (I-P, a health care professional).^40^^(p.5)^
		Approach to transition (U)	It’s very tick-the-box. (A health care professional)^55^^(p.787)^
		The children’s model is different to the adult model (U)	The paediatric model is quite different from the adult model of care in that if they don’t come to clinic…we chase them, whereas in the adult field…it is up to them to do the chasing…the adult model is…here is the information, do with it what you will. In the paediatric scene…we don’t…give up on them…they’re babied you know. (A paediatrician)^51^^(p.766)^
		Lack of psychology service (U)	You will know medically what is going on with them but from a psychological point of view it is the joint paediatric-adult ANP that helps with that. (A health care professional)^55^^(p.787)^
		Attendance (U)	You have a 50% did not attend rate. (A health care professional)^55^^(p.787)^
		Working with what is available (C)	… the majority of their [General Practitioner] caseload is older people with diabetes …. where it’s so much about complications management. Their lifestyle advice that they are used to giving is not lifestyle advice that suits a teen or young adult lifestyle. (HCP:1)^39^^(p.338)^
		Ending relationship in paediatric care (U)	The young man came with his father for his first visit to adult diabetes care. He told me himself that they had already talked about it when he was 18 that he would transfer, but that there could be a long waiting time. When he hadn’t been given an appointment for 1.5 years, he rang to paediatrics himself because he needed a new prescription. (O-A, observation filed notes)^40^^(p.5)^
		Timing (U)	There’s a lot of change in a young person’s life - going to college, leaving home…makes it even more difficult. (A health care professional)^55^^(p.787)^
	HCPs’ suggestions to improve transition	Preparation (U)	You let them know that they’ll be moving on. (A health care professional)^55^^(p.787)^
		More structured processes (U)	I think we need to start our young people early and let them know and let them get excited about transitioning themselves. (Diabetes educator)^53^^(p.114)^
		Adjustment of strategies (U)	It would probably be good for the patients, to prepare and for us to know what they have been used to before, because we actually don’t know that, well I don’t anyway. (I-A, a health care professional)^40^^(p.5)^
		Education (U)	Every consultation, alcohol is talked about with every single young adult… same with smoking. (A health care professional)^55^^(p.787)^
		Key person (U)	A is the paediatric and the adult nurse. (A health care professional)^55^^(p.787)^
		Mapping a route to better services (U)	I think if you really want to target young people with type 1 diabetes, you do not say that an 18-year-old is the same as a 30-year-old. I think they need separate group sessions where you can target the different age ranges or different developmental stages. (HCP:6)^39^^(p.339)^
		Bridging activities (U)	It is important for us to hear how it has … what affects the adolescent and what their weaknesses … you can’t just say that they have neglected themselves but there are a number of problems which they have no control over …which makes it difficult and you should have an understanding of this as well when they come. (I-A, a health care professional)^40^^(p.5)^
		Introducing adult care (U)	The nurse, working in both settings, gives the phone number at both places and says that he will be given an appointment in September and perhaps to day-care group if he is interested sometime next year. (O-P, observation filed notes)^40^^(p.6)^
		Exploring knowledge and motivation (U)	It’s not really the knowledge that is lacking, but the motivation to self manage and understand … there are so many other things at this age which is important to them. (I-P, a health care professional)^40^^(p.6)^
		Increasing contact desired (U)	I think it’s good, now we get a totally different offer from the PDC thanks to the new nurse from paediatrics… it gives us so much more, there are so many things that we don’t always think about on the adult side and vice versa… we have already understood that the nurse has implemented certain things that no one thought about before. (I-A, a health care professional).^40^^(p.6)^
		Improving strategies after feedback (U)	It is nevertheless quite encouraging if you find out that something of what you did has stuck … and that they have benefited from the support they received then, maybe it will turn out better in the long-term anyway …so it’s very important I think. (I-P, a health care professional).^40^^(p.6)^
		Expanding the role played by primary care physicians (U)	Where it’s easier for them [the patient] to get in to see maybe their [primary care physician], as opposed to coming in to see the [diabetes care] team… that would be a really great way to keep them connected with the health care system. (Paediatrician)^53^^(p.114)^
		Referral (U)	Discharge summary from paeds… tells you everything you need to know about them. (A health care professional)^55^^(p.787)^

U, unequivocal; C, credible

ANP, advanced nurse practitioner; GP, general practitioner; HCP, health care provider; PDC, pediatric diabetes clinic; T1D, type 1 diabetes.
